# Biophysical determinants of membranotropism: how proteins and peptides recognize, insert into, and remodel lipid bilayers

**DOI:** 10.1007/s12551-026-01464-5

**Published:** 2026-09-25

**Authors:** Nadine Lobo Siqueira, Yhony Sumari Misaico, Thais Rangel Figueiredo, Miguel Eduardo Salazar Aurich, Amanda Toledo Machado, Lorran Yves Machado de Sousa, Fadi Simon de Souza Magalhães, Ana Eliza Zeraik, Luis Guilherme Mansor Basso

**Affiliations:** 1 Laboratório de Química e Função de Proteínas e Peptídeos, Universidade Estadual do Norte Fluminense Darcy Ribeiro, Avenida Alberto Lamego, 2000, Campos dos Goytacazes, RJ 28013-602 Brazil; 2 Laboratório de Ciências Físicas, Centro de Ciência e Tecnologia, Universidade Estadual do Norte Fluminense Darcy Ribeiro, Avenida Alberto Lamego, 2000, Campos dos Goytacazes, RJ 28013-602 Brazil

**Keywords:** Membranotropism, Protein–membrane interactions, Membrane biophysics

## Abstract

Biological membranes are not passive barriers, but chemically heterogeneous and physically adaptable interfaces that regulate how proteins and peptides bind, fold, assemble, and act. Many membrane-active systems cannot be adequately described as simply soluble, peripheral, or transmembrane. Instead, they populate multiple membrane-associated states, ranging from transient surface adsorption to interfacial partitioning, insertion, oligomerization, pore formation, fusion, and large-scale membrane remodeling. In this review, we discuss membranotropism as a unifying concept to describe the propensity of proteins, peptides, and supramolecular assemblies to associate with lipid bilayers and modify their structure, organization, or function. We bring together biophysical principles that connect membrane composition, lipid packing, curvature, sterol availability, lateral heterogeneity, oxidative state, molecular crowding, and membrane mechanics with molecular features such as amphipathicity, charge distribution, conformational plasticity, lipid-recognition motifs, and cooperative assembly. This conceptual perspective is illustrated with examples that include antimicrobial and antifungal peptides, viral fusion peptides, pore-forming toxins, peripheral and amphitropic proteins, monotopic enzymes, septin scaffolds, secreted membrane modulators, and condensate–membrane systems. We also highlight how complementary experimental and computational approaches can resolve membrane binding, partitioning, insertion, remodeling, and functional membrane activity across different length and time scales. By integrating molecular architecture with membrane biophysics, membranotropism provides a useful perspective for understanding how membrane recognition is converted into biological activity. We also discuss how this concept can guide the development of antimicrobial, antiviral, antiparasitic, anticancer, and delivery-oriented strategies based on the selective targeting or remodeling of lipid membranes.

## Introduction

### Biological membranes as dynamic interaction platforms

Biological membranes are essential boundaries that separate the cellular interior from the external environment while simultaneously acting as dynamic platforms for molecular recognition, signaling, transport, and energy transduction. Rather than passive barriers, membranes should be viewed as chemically diverse and physically adaptable interfaces whose properties emerge from the cooperative behavior of lipids, proteins, and other membrane-associated molecules. This functional complexity is supported by the remarkable diversity of cellular lipids, which contributes to the modulation of membrane fluidity, permeability, curvature, thickness, charge, and lateral organization (van Meer et al. 2008; Harayama and Riezman 2018).

Lipids not only form the structural matrix of the bilayer but also establish essential interactions with proteins, providing a suitable environment for the anchoring, folding, organization, and regulation of membrane-associated proteins (Corradi et al. 2019). Considering that transmembrane proteins represent approximately 30% of the mammalian proteome and play central roles in signaling, transport, metabolism, and cellular communication, many cellular processes rely on the intimate interplay between membrane lipids and proteins (Harayama and Riezman 2018). Although membrane functions have historically been attributed mainly to proteins, it is now clear that lipids actively participate in regulating protein structure, dynamics, localization, and activity (Corradi et al. 2019).

The properties exerted by lipids depend directly on their chemical structures, including the nature of their polar head groups, the length and degree of saturation of acyl chains, and the presence of sterols or charged groups. These structural variations determine essential biophysical parameters, such as lipid packing, bilayer thickness, membrane curvature, lateral mobility, surface charge, and the propensity to form lamellar or nonlamellar phases. Consequently, changes in lipid composition can directly influence protein binding, insertion, oligomerization, conformational equilibria, and the formation of membrane microdomains (Harayama and Riezman 2018). In this sense, membranes behave as cooperative systems in which lipid-lipid, protein–protein, and lipid-protein interactions (Nicolson 2014) are interconnected and collectively define membrane organization and function. In this cooperative context, lipid composition defines the local bilayer environment encountered by proteins and peptides, whereas membrane-associated molecules can reciprocally reorganize lipids, stabilize specific membrane states, or assemble into functional complexes (van Meer et al. 2008; Quinn 2012; Walker et al. 2024). Thus, membrane function emerges from the dynamic coupling between lipid organization and protein architecture, rather than from either component alone.

The importance of lipid–protein interactions has been recognized for decades. Early studies on the effects of cholesterol on rhodopsin (Mitchell et al. 1990; Albert et al. 1996), bilayer composition and fluidity on gramicidin assembly and ion transport (O’Connell et al. 1990), and lipid bilayer thickness on conformational transitions of the Ca^2+^-ATPase calcium pump (Lee 1998) helped establish the concept that the lipid environment plays an active role in the structural and functional modulation of membrane proteins. These examples illustrate a broader principle: membrane composition and physical state are not merely background variables, but determinants of protein energetics, dynamics, and function. Understanding how membrane properties influence protein and peptide behavior has therefore become a major driving force in modern biophysics, particularly at the interface between structure, dynamics, and biological activity (Lee 2011).

This perspective is especially relevant for proteins and peptides that interact with membranes without necessarily behaving as classical transmembrane proteins. Antimicrobial peptides, toxins, viral fusion peptides, peripheral signaling proteins, curvature-sensing domains, cholesterol-binding motifs, intrinsically disordered regions, and secreted membrane-active proteins can recognize, bind, insert into, remodel, or disrupt lipid bilayers through distinct but often overlapping mechanisms. These systems highlight the need for an integrative concept to describe how structural features such as charge distribution, hydrophobicity, amphipathicity, secondary-structure propensity, aromatic residues, oligomerization, and conformational plasticity determine the tendency of proteins and peptides to associate with lipid membranes. In this review, we refer to this tendency as membranotropism.

### Defining membranotropism: a continuum of membrane interaction states

At the molecular level, the tendency described above is commonly discussed under the broader expression *membrane interaction*, which encompasses the physical association and molecular contacts established between biological membranes and molecules, peptides, proteins, domains, or supramolecular assemblies (Upton et al. 2024). Such interactions may involve specific headgroup recognition, electrostatic attraction at the membrane surface, interfacial partitioning, partial or deep insertion into the bilayer, hydrophobic matching, or shape complementarity (Hurley 2006). Although *membrane interaction* remains the most widely used expression, the terms *membranotropism* and *membranotropic* have also been used to describe the ability of molecules, peptides, proteins, or supramolecular assemblies to associate with membranes and modulate their properties (Azouzi et al. 2015; Basso et al. 2021; Ghazaryan et al. 2015; Nemesio et al. 2012; Walrant et al. 2017).

The term tropism has been used in different biological contexts and acquires specific meanings depending on the system under consideration. It was originally associated with directional plant responses to external stimuli, such as gravity (*gravitropism*), light (*phototropism*), moisture (*hydrotropism*), or temperature (*thermotropism*) (Mast 1915; Bisgrove 2008). In infectious diseases, pathogen or viral tropism describes the preference of an infectious agent for particular cell types, tissues, or host species (McFadden et al. 2009; McCall 2021). More broadly, tropism can be understood as the tendency of a system to respond in a defined manner to a specific stimulus, environment, or biological context. By extension, *membranotropism* may be defined as the propensity of a molecule or molecular assembly to associate with a lipid membrane, or with specific membrane components, through structurally and energetically favorable interactions that result in measurable binding, insertion, reorganization, or functional modulation of the membrane.

In this sense, membranotropism is not intended to replace established terms such as membrane binding, membrane interaction, or membrane activity. Rather, it provides an integrative concept that connects these terms across different levels of description. Membrane binding refers primarily to the association of a molecule with a membrane and is often described in terms of affinity, partitioning, residence time, or membrane-associated fraction. Membrane interaction refers more broadly to the physicochemical contacts established at the membrane surface or within the bilayer, including electrostatic attraction, lipid recognition, interfacial anchoring, hydrophobic insertion, or hydrophobic matching. Membrane activity refers to the functional consequence of these interactions, such as permeabilization, fusion, curvature generation, signaling modulation, lipid reorganization, or membrane disruption. Membranotropism emphasizes the propensity of a molecule or assembly to recognize particular membrane environments, populate specific membrane-associated states, and convert molecular architecture and membrane physicochemistry into structural or functional outcomes. Thus, the term is used here as a conceptual framework that links membrane selectivity, interaction mode, and biological consequence, rather than as a synonym for any single one of these processes.

Membranotropic agents can therefore be broadly understood as molecules or assemblies whose biological or physicochemical activity depends on their interaction with lipid bilayers. Their behavior is governed by the interplay between molecular architecture and membrane properties. Important determinants include molecular geometry, hydrophobicity, amphipathicity, dipole moment, charge distribution, conformational plasticity, and the spatial arrangement of polar, nonpolar, and aromatic groups. Accordingly, membranotropism emerges from the combined contribution of these structural parameters and from the physicochemical state of the target membrane, including lipid composition, charge, packing, curvature, thickness, and phase behavior. Although the term has been frequently applied to membranotropic peptides, including antimicrobial peptides, cell-penetrating peptides, viral fusion peptides, anticancer peptides, amyloidogenic peptides, and toxins (Galdiero et al. 2013), it can also be extended to membrane-binding domains (Hurley 2006; Vicente et al. 2017), peripheral proteins (Sastre et al. 2020a), secreted membrane-active proteins (Felizatti et al. 2020), amphipathic drugs (Lucio et al. 2010), surfactants (Otzen 2017), lipophilic natural compounds (Tsuchiya 2015), and nanoparticles (Izzi et al. 2023).

Accordingly, throughout this review, membranotropism is discussed as a continuum of membrane-associated states that connects membrane recognition, interaction mode, and functional outcome. These states may range from transient surface adsorption to stable interfacial partitioning, shallow or deep insertion, membrane-induced folding, oligomerization, pore formation, curvature generation, fusion, translocation, or large-scale membrane disruption. This continuum constitutes the conceptual framework used here to understand how different protein and peptide architectures engage lipid bilayers, how membrane binding progresses into distinct interaction states, and how these states are translated into membrane remodeling and biological function.

## Membrane determinants of selectivity and specificity

Lipid–protein interactions range from highly specific recognition of individual lipid species by defined protein-binding sites to more general effects mediated by the physical properties of the membrane. In this context, membrane lipids can be broadly classified as bulk lipids, annular, or non-annular lipids. Bulk lipids do not directly bind to membrane proteins but influence their geometry, dynamics, localization, and activity through collective membrane properties (He et al. 2025). Annular lipids form a boundary shell around transmembrane domains, whereas non-annular lipids interact with specific protein sites and may stabilize particular conformational or functional states (He et al. 2025; Anderluh et al. 2017). Thus, membrane selectivity arises not only from direct molecular recognition but also from the capacity of proteins and peptides to sense distinct bilayer physical properties. In cellular membranes, these lipid-dependent determinants are further shaped by molecular crowding, glycoconjugates, protein density, and other features of the native membrane interface that are only partially captured by simplified model systems.

### Lipid composition, charge, and curvature

The physicochemical properties of membrane lipids are fundamental determinants of membrane organization and protein activity. Variations in lipid headgroups, acyl-chain length, unsaturation, charge, and sterol content modulate bilayer thickness, fluidity, packing, surface potential, and curvature stress. These parameters define the energetic landscape encountered by membranotropic proteins and peptides and strongly influence their adsorption, insertion, folding, oligomerization, and functional activity (Teixeira et al. 2012). Although the full morphological complexity of cellular membranes usually requires the coordinated action of lipids, proteins, and the cytoskeleton, specific lipid compositions can induce membrane deformation, budding, tubulation, and domain formation in model systems such as giant unilamellar vesicles (Lingwood and Simons 2010).

Membrane curvature is particularly relevant for membrane-active systems because it alters lipid packing, leaflet asymmetry, and the exposure of hydrophobic defects. The spontaneous curvature of a lipid is determined by its molecular geometry and by interactions with neighboring lipids within a leaflet. Lipids with relatively large polar headgroups and smaller hydrophobic regions, such as lysophospholipids, tend to favor positive curvature, whereas cone-shaped lipids with smaller headgroups or larger hydrophobic regions, such as phosphatidylethanolamine, phosphatidic acid, and diacylglycerol, favor negative curvature and can stabilize invaginated, hemifusion, or non-lamellar intermediates (Seddon 2025). More cylindrical lipids, such as phosphatidylcholine and phosphatidylserine, generally favor relatively flat bilayers (Seddon 2025). Curvature can also arise through bilayer-couple effects, in which lipid redistribution or asymmetric insertion of molecules into one leaflet generates differential leaflet expansion and membrane bending (Vanni et al. 2014; Vamparys et al. 2013).

Curvature stress can influence membranotropism by changing the energetic cost of membrane insertion and remodeling. Highly curved or stressed regions often contain packing defects that expose hydrophobic portions of the bilayer to the aqueous environment, facilitating the insertion of amphipathic peptides, protein domains, or hydrophobic loops. As a result, curvature can promote membrane thinning, local disorder, transient permeabilization, pore formation, fusion, or transitions toward non-lamellar phases (McMahon and Gallop 2005; Teixeira et al. 2012). Conversely, membrane-active proteins and peptides can reshape membranes by stabilizing or amplifying curvature, creating a reciprocal relationship between lipid geometry and molecular architecture.

Lipid oxidation represents another important compositional modification that can reshape the membrane environment encountered by proteins and peptides. Polyunsaturated acyl chains are particularly susceptible to enzymatic or reactive oxygen species-mediated oxidation, generating lipid hydroperoxides, truncated phospholipids, and secondary reactive products such as aldehydes (Fruhwirth et al. 2007; Bochkov et al. 2010). The introduction of oxygen-containing groups into acyl chains can promote their reorientation toward the membrane–water interface, disrupting lipid–lipid interactions, increasing interfacial hydration, modifying packing defects, reducing bilayer thickness and order, and altering membrane permeability (Wong-Ekkabut et al. 2007). Oxidized phospholipids may also modify lipid geometry, spontaneous curvature, bending behavior, and, in some cases, interfacial charge distribution, with effects that depend on membrane composition and on the specific oxidation products generated (Mosca et al. 2011; Jin and Baumgart 2021; Drabik 2026). These changes can directly influence membranotropism by altering the energetic cost of adsorption, insertion, folding, and aggregation at the membrane interface. For example, oxidation of ω−6 polyunsaturated fatty acyl chains can generate 4-hydroxy-2-nonenal, a reactive aldehyde that covalently modifies histidine residues in amyloid-β peptides through Michael addition, increasing peptide membrane affinity, local concentration at the membrane surface, and fibrillogenesis (Liu et al. 2008). Thus, oxidized lipids and lipid oxidation products provide a disease-relevant layer of membrane selectivity in conditions associated with oxidative stress, inflammation, atherosclerosis, ferroptosis, and protein aggregation (Bochkov et al. 2010; Do and Xu 2023).

Charged lipids also provide an important source of selectivity. Anionic lipids, such as phosphatidylserine, phosphatidylglycerol, phosphatidic acid, and phosphoinositides, create electrostatic fields that favor the recruitment of basic residues and polycationic domains. Phosphoinositides are especially important regulatory lipids despite their relatively low abundance (Gamper and Shapiro 2007). Phosphatidylinositol is the precursor of phosphoinositides and accounts for approximately 5–8% of total cellular lipids; phosphorylation at different positions of the inositol ring generates distinct phosphoinositide species that are differentially enriched in cellular membranes (van Meer et al. 2008). Among them, PI(4,5)P_2_ is abundant in the cytoplasmic leaflet of the plasma membrane and acts both as a structural membrane component and as a signaling lipid (Katan and Cockcroft 2020; McLaughlin and Murray 2005). PI(4,5)P_2_ illustrates how a minor lipid species can exert strong regulatory effects on membrane-associated proteins, for example by binding basic regions of vinculin to promote conformational opening, oligomerization, focal adhesion strengthening, and association with actin filaments (Chinthalapudi et al. 2014) or by stabilizing oligomeric states of the human serotonin transporter at the plasma membrane (Anderluh et al. 2017). These examples show that lipid composition, oxidative state, charge, and curvature do not act independently but jointly define membrane environments that selectively recruit, stabilize, or modulate membranotropic proteins and peptides.

### Sterols as determinants of membrane recognition

Sterols are major regulators of membrane organization and selectivity. Cholesterol modulates bilayer order, fluidity, thickness, permeability, and the formation of specialized membrane domains. Its enrichment in animal cell membranes also provides a molecular signature that distinguishes host membranes from many bacterial, fungal, or plant membranes, which are enriched in hopanoids, ergosterol, or phytosterols, respectively (Rothman and Lenard 1977; Heuck et al. 2010). Therefore, sterol identity and availability can determine whether a membrane is recognized, remodeled, or disrupted by a given protein or peptide.

Cholesterol-dependent cytolysins (CDCs) provide a clear example of sterol-dependent membrane specificity. These bacterial β-pore-forming toxins (β-PFTs) selectively bind cholesterol-rich eukaryotic membranes (Morton et al. 2019). In the clostridial toxin perfringolysin O (PFO), cholesterol recognition involves a conserved cholesterol-recognition motif containing threonine and leucine residues (T490 and L491); mutation of these residues impairs membrane binding and pore formation. After cholesterol-dependent binding, toxin monomers oligomerize into circular structures known as prepore complexes and undergo conformational rearrangements that insert β-hairpins into the bilayer to form membrane pores (Morton et al. 2019; Farrand et al. 2010; van Pee et al. 2016).

Importantly, cholesterol recognition depends not only on total cholesterol concentration but also on cholesterol accessibility. Phosphatidylcholine-rich membranes can keep cholesterol more deeply buried, limiting protein access, whereas phosphatidylethanolamine and sphingomyelin can modify cholesterol exposure, domain organization, and toxin binding efficiency (Morton et al. 2019; Simons and Ikonen 1997). Thus, the functional pool of cholesterol available for protein recognition may differ substantially from the total sterol content of the membrane.

A related example is the Hedgehog (Hh) signaling pathway, in which cholesterol accessibility, rather than total membrane cholesterol, regulates membrane-dependent signaling. In vertebrates, Sonic Hedgehog (SHH) signals through Patched 1 (PTCH1) and smoothened (SMO), but PTCH1 and SMO do not appear to interact directly. Current models propose that PTCH1 limits the pool of accessible cholesterol available to activate SMO; upon SHH binding, PTCH1 inhibition increases accessible cholesterol and promotes SMO activation (Kong et al. 2019; Radhakrishnan et al. 2020). Overall, these examples show that sterol-dependent membranotropism is determined not only by sterol identity or abundance, but also by its accessibility and organization within the bilayer.

### Membrane phase and lateral heterogeneity: Lo versus Ld states

Membrane selectivity also depends on lateral heterogeneity. Biological membranes can contain ordered and disordered regions that differ in lipid composition, packing, thickness, permeability, mechanical properties, and protein affinity (Sezgin et al. 2017). In model membranes, mixtures containing saturated lipids, unsaturated phospholipids, and cholesterol can undergo lateral phase separation into liquid-ordered (Lo) and liquid-disordered (Ld) phases. The Lo phase is enriched in sterols and saturated lipids and combines high acyl-chain order with lateral fluidity, making it a simplified model for lipid raft-like domains (Kaiser et al. 2009; Sezgin et al. 2017).

Phase separation is not only a compositional phenomenon, but also a mechanical one. Together with bilayer leaflet asymmetry, lateral segregation can generate line tension, membrane stress, and domain boundaries that facilitate local deformation and influence protein recruitment (Lingwood and Simons 2010). Many membrane-associated proteins interact preferentially with specific lipid species or physical states, particularly those enriched within ordered or raft-like domains. As a result, lipid composition and phase behavior can regulate the recruitment, distribution, residence time, and dissociation of membrane-active proteins, thereby influencing both membrane organization and cellular function.

Lipid raft-like domains can concentrate or exclude specific proteins, contributing to signaling, trafficking, recognition, and membrane remodeling. However, these domains should not be viewed as rigid or homogeneous barriers. Recent studies suggest that Lo phases may exhibit mosaic-like organization, with compact saturated-lipid regions separated by cholesterol- and unsaturated-lipid-enriched regions. Such organization can reduce permeability to small molecules relative to Ld phases while still allowing selective diffusion through specific membrane regions (Ghysels et al. 2019). Therefore, phase sensitivity provides another layer of membranotropism, in which proteins and peptides respond not only to lipid composition, but also to nanoscale organization, packing defects, membrane thickness, and domain boundaries.

Together, lipid composition, charge, curvature, sterol accessibility, and membrane phase behavior define key layers of the selectivity landscape encountered by membrane-active proteins and peptides. In cellular membranes, however, these physicochemical determinants operate within a crowded and molecularly complex environment, as discussed below.

### Membrane crowding and molecular complexity

Biological membranes are highly crowded and heterogeneous environments in which proteins, lipids, glycoconjugates, and other membrane-associated macromolecules coexist within a limited interfacial area. Estimates of membrane protein densities indicate that cellular membranes may contain approximately 30,000 proteins per µm^2^, leaving only a few nanometers of available space between neighboring proteins (Guigas and Weiss 2016). Such molecular crowding distinguishes biological membranes from the simplified lipid bilayers commonly used in biophysical studies and introduces steric, entropic, and thermodynamic constraints that can influence membrane-associated processes. In particular, high protein densities can increase the probability of nonspecific encounters, restrict available membrane area, and affect the organization and dynamics of both proteins and lipids within the membrane (Löwe et al. 2020).

Membrane crowding can consequently modulate the lateral mobility, spatial organization, and conformational behavior of membrane-associated molecules. Molecular simulations and experimental studies have shown that increasing protein density can reduce the lateral diffusion of both membrane proteins and neighboring lipids, while generating heterogeneous and anomalous diffusion regimes (Goose and Sansom 2013; Jeon et al. 2016). Crowding can also promote or stabilize protein clustering and influence the nanoscale organization of membrane proteins, with its effects being coupled to lipid composition and membrane complexity (Duncan et al. 2017). From a thermodynamic perspective, crowding within the two-dimensional membrane environment can influence the effective activity, orientation, redistribution, and intermolecular association of membrane-associated proteins and peptides, highlighting that membrane organization cannot be understood solely from isolated protein–lipid interactions (Zhou 2009).

These effects are particularly relevant to membranotropic peptides and proteins, for which membrane association may be followed by interfacial partitioning, insertion, oligomerization, or membrane remodeling. The local density and organization of pre-existing membrane components may influence bilayer accessibility, lateral mobility, residence time, and the probability of subsequent intermolecular interactions (Löwe et al. 2020, 2023; Zhou 2009). The effective behavior of a membranotropic molecule may therefore depend not only on its intrinsic affinity for lipids, but also on the molecular context in which membrane association occurs (Zhou 2009; Guigas and Weiss 2016). This consideration is especially important when interpreting experiments performed in simplified lipid bilayers, which are valuable for resolving molecular mechanisms but may not reproduce the high protein density, glycocalyx, and spatial heterogeneity of cellular membranes (Löwe et al. 2020, 2023; Schmitter et al. 2026). Recent studies have also introduced experimental approaches for probing crowding at membrane interfaces, including genetically encoded fluorescence-based sensors validated in both reconstituted and cellular membrane systems (Löwe et al. 2023; Schmitter et al. 2026). Therefore, membrane crowding should be considered an additional contextual determinant of membranotropism, linking the intrinsic properties of membrane-active molecules to the complex physicochemical environment of biological membranes.

## Structural and thermodynamic determinants of membranotropism

Having considered how membrane composition, charge, curvature, sterol content, lateral organization, and molecular crowding influence selectivity, we now shift the focus to the molecular determinants that allow proteins and peptides to recognize, bind, insert into, and remodel lipid bilayers. Membranotropism cannot be predicted from bulk hydrophobicity alone but depends on the three-dimensional distribution of charge, hydrophobicity, amphipathicity, secondary-structure propensity, conformational plasticity, aromatic residues, and specific lipid-recognition motifs (White and Wimley 1999; von Heijne 2006). These features determine how a molecule samples the membrane–water interface, partitions into the bilayer, undergoes membrane-induced folding, and couples binding to functional membrane perturbation.

Membrane association is therefore rarely a single binding event. Membrane-active peptides, proteins, and amphiphilic molecules often populate a sequence of coupled states, including surface adsorption, interfacial partitioning, conformational rearrangement, partial or deep insertion, oligomerization, and perturbation of lipid organization (Avci et al. 2018; Guha et al. 2019). Although these events are frequently represented as discrete steps, they may occur concomitantly and influence one another. Thus, membrane “binding” should be interpreted as an apparent outcome of multiple energetic contributions, including electrostatic attraction, dehydration, hydrophobic partitioning, hydrogen bonding, conformational changes, and lipid reorganization (Seelig 2004). This section examines how structural motifs and thermodynamic forces combine to define the membrane-associated states of proteins and peptides.

### Structural determinants of membrane recognition and insertion

#### Charge distribution and electrostatic interactions

Electrostatic interactions are among the first determinants governing the approach of proteins and peptides to membranes. The membrane–water interface is characterized by a steep polarity gradient, with a high dielectric constant in bulk water (~ 80) and a much lower dielectric constant within the hydrocarbon core (~ 2) (McLaughlin 1989). This anisotropic environment strongly affects the partitioning of charged and polar groups. Anionic lipids such as phosphatidylserine, phosphatidylglycerol, phosphatidic acid, and phosphoinositides create negatively charged surfaces that favor the recruitment of basic residues and polycationic domains. Under physiological ionic strength, these long-range electrostatic interactions are partially screened, but they remain important for increasing local concentration, orienting membrane-active molecules at the interface, and facilitating subsequent hydrophobic insertion or folding transitions (McLaughlin 1989; Corradi et al. 2018).

In integral membrane proteins, charge distribution also contributes to topology (von Heijne 1986). The “positive-inside rule” describes the enrichment of arginine and lysine residues in cytoplasmic loops connecting transmembrane helices, helping determine the orientation of membrane insertion (von Heijne 1986; 1992). This rule reflects the energetic cost of transferring charged groups across the hydrophobic core and the favorable stabilization of basic residues at the membrane interface. During membrane insertion, the Sec61 translocon can be viewed as a molecular environment that samples the energetic compatibility of nascent hydrophobic segments with the bilayer (Rapoport 2007). Thus, the distribution of charged residues provides both topological information and interfacial anchoring.

Although arginine (Arg) and lysine (Lys) are both positively charged residues, they behave differently in membrane environments. The primary thermodynamic barrier to translocating an unshielded charge into the low-dielectric aliphatic core of the lipid bilayer is the substantial dehydration penalty, a free energy cost mathematically governed by the Born solvation energy model (Choe 2024; Dorairaj and Allen 2007).

Lysine can undergo environment-dependent protonation changes, which may facilitate deeper partitioning under some conditions (MacCallum et al. 2008). Arginine, by contrast, usually maintains its charge because of the resonance-stabilized guanidinium group (Yoo and Cui 2008; Dorairaj and Allen 2007). This group forms multiple hydrogen bonds and strong electrostatic interactions with phosphate headgroups and interfacial water, often stabilizing membrane defects and promoting local bilayer deformation (Rothbard et al. 2004). Both residues can undergo “snorkeling,” in which their aliphatic portions interact with the hydrocarbon region while the terminal charged group remains near the polar interface, stabilizing transmembrane or interfacial segments and helping compensate for hydrophobic mismatch (Kandasamy and Larson 2006). Collectively, these principles show that charge distribution influences not only the initial electrostatic recruitment to membranes, but also molecular orientation, insertion depth, and stabilization at the membrane interface.

#### Hydrophobicity, amphipathicity, and interfacial partitioning

Hydrophobicity is a central determinant of membrane association, but global hydrophobicity alone is insufficient to predict membranotropism. The spatial organization of hydrophobic and hydrophilic residues is often more informative than their total abundance. Amphipathic structures, particularly α-helices, display hydrophobic and polar/charged residues on opposite faces, allowing simultaneous interaction with the lipid acyl region and the aqueous or headgroup environment. The hydrophobic moment provides a useful measure of this asymmetric residue distribution and helps distinguish interfacial amphipathic helices from more uniformly hydrophobic transmembrane segments (Eisenberg et al. 1982).

Amphipathic helices often bind parallel to the membrane surface, inserting bulky hydrophobic side chains shallowly into one leaflet while maintaining polar and charged residues at the interface (Drin and Antonny 2010). This mode of interfacial partitioning is favored by lipid-packing defects, which expose hydrophobic regions of the bilayer to the aqueous phase and lower the energetic cost of insertion. Aromatic residues such as tryptophan and tyrosine can further stabilize interfacial binding by anchoring peptides or protein segments near the headgroup–acyl-chain boundary. Thus, amphipathicity does not simply promote membrane binding; it defines how deeply and asymmetrically a molecule partitions into the bilayer interface.

This shallow and asymmetric insertion can directly affect membrane mechanics. By locally displacing phospholipid headgroups and increasing the effective surface area of one leaflet relative to the other, amphipathic helices alter the lateral pressure profile and generate curvature stress (Bigay et al. 2005; Campelo et al. 2008; McMahon and Gallop 2005). In this sense, amphipathicity links molecular structure to membrane remodeling: the same residue distribution that favors interfacial partitioning can also induce lipid disorder, leaflet asymmetry, curvature generation, and functional membrane deformation.

#### Secondary-structure propensity and membrane-induced folding

The low dielectric interior of lipid bilayers imposes a high energetic penalty on unpaired polar backbone groups. For this reason, membrane insertion often requires the formation of secondary structures that internally satisfy backbone hydrogen-bonding requirements. Transmembrane α-helices are particularly favorable because their regular intramolecular hydrogen-bonding pattern shields backbone dipoles while allowing extensive van der Waals contacts with lipid acyl chains (Engelman et al. 1986; Palczewski et al. 2000). However, membrane helices are not necessarily rigid rods. Conserved proline and glycine residues can introduce kinks, hinges, or local flexibility, enabling conformational transitions required for gating, transport, signaling, and allosteric regulation (Papadopoulou et al. 2022).

β-structured membrane-active systems provide an alternative solution to the same energetic problem. In bacterial, mitochondrial, and chloroplast outer membranes, β-barrel proteins satisfy backbone hydrogen bonding through antiparallel β-strands that curve into closed cylindrical structures, exposing hydrophobic residues to the lipid environment and forming internal aqueous pores (Vorobieva et al. 2021). Smaller peptides, including β-hairpin antimicrobial peptides and toxins, can use alternating hydrophobic and hydrophilic residues to generate planar amphipathic surfaces. Upon membrane binding, these structures may adsorb obliquely, self-associate, and form oligomeric assemblies associated with membrane permeabilization or pore formation (Fahrner et al. 1996). Thus, both α-helical and β-structured architectures solve the thermodynamic challenge of membrane association by coupling backbone stabilization to lipid-facing amphipathicity.

Intrinsically disordered regions (IDRs) add another layer of complexity. Many membrane-associated proteins contain disordered segments that lack stable structure in solution but undergo disorder-to-order transitions upon binding to membranes. This folding-upon-binding mechanism can increase capture efficiency, allow multivalent interactions, and generate amphipathic helices or other transient structures at the interface (Wimley 2010). IDRs may also sense membrane curvature through entropic mechanisms, preferentially associating with highly curved surfaces where conformational restriction is reduced (Zeno et al. 2018). Because of their flexibility and multivalency, IDRs may also contribute to weak, transient interactions at membranes and to the coupling between membrane surfaces and biomolecular condensates (Ray et al. 2020). Thus, membrane association can be supported either by preorganized secondary structures or by membrane-induced folding, highlighting conformational plasticity as a key determinant of membranotropism.

#### Specific structural motifs and membrane-recognition modules

In addition to general physicochemical determinants, many proteins use defined membrane-binding domains or short linear motifs to achieve selectivity. These modules often operate through coincidence detection, in which simultaneous recognition of multiple membrane features, such as charge, curvature, lipid identity, or calcium binding, generates sufficient free energy to recruit a protein to a specific membrane compartment. This modular strategy allows membrane targeting to be both selective and reversible.

C2, PH, and BAR domains illustrate distinct modes of membrane recognition. C2 domains, such as those found in synaptotagmin, often use Ca^2+^ binding as an electrostatic switch that promotes association with anionic membranes (Zhou et al. 2017). PH domains recognize phosphoinositide headgroups through positively charged binding pockets, thereby targeting proteins to membranes enriched in specific phosphoinositide species, such as PI(4,5)P_2_ or PI(3,4,5)P_3_ (Thomas et al. 2002). BAR domains use curved, positively charged surfaces to bind anionic membranes and impose or stabilize membrane curvature (Gallop et al. 2006). Together, these domains show how proteins can translate lipid composition and membrane geometry into spatially regulated recruitment and remodeling.

Short motifs can also mediate specific lipid recognition. Cholesterol-recognition motifs, including CRAC and CARC sequences, are found in several transmembrane proteins and have been proposed to contribute to cholesterol binding, protein localization, oligomerization, and allosteric regulation (Gallop et al. 2006; Di Scala et al. 2017). Although their predictive value can depend on structural context, these motifs highlight how sterol recognition may be encoded within transmembrane or juxtamembrane segments (Fantini and Barrantes 2013).

Aromatic residues, particularly tryptophan and tyrosine, are also enriched at membrane interfaces, forming so-called aromatic belts around transmembrane segments. Their interfacial preference reflects their ability to combine hydrophobic partitioning, hydrogen bonding, dipole interactions, and cation–π interactions with lipid headgroups or neighboring residues (Braun and von Heijne 1999). Aromatic residues therefore act as interfacial anchors that stabilize membrane-associated structures, define insertion depth, and modulate conformational transitions.

### Thermodynamic landscape of membrane association

Membranotropism cannot be fully described by a single binding constant or by a single thermodynamic signature. Membrane-active molecules usually populate a sequence of coupled states, including electrostatic surface enrichment, interfacial partitioning, membrane-induced folding, shallow or deep insertion, oligomerization, and membrane perturbation. Each of these steps may involve distinct energetic contributions, and the apparent thermodynamic parameters measured experimentally depend on which molecular state is being populated and how the membrane-associated state is defined.

Membrane partitioning provides a useful quantitative description of the equilibrium between aqueous and membrane-associated populations. In its most general form, a partition coefficient expresses the ratio between the concentration of a peptide or protein in the membrane-associated state and its free concentration in the aqueous phase, ${K}_{p}=({\left[P\right]}_{\mathrm{m}\mathrm{e}\mathrm{m}\mathrm{b}}/{\left[P\right]}_{\mathrm{f}\mathrm{r}\mathrm{e}\mathrm{e}})$. This definition is conceptually analogous to classical partitioning between two phases, but lipid bilayers are not homogeneous bulk solvents. Instead, they are anisotropic interfacial environments composed of lipid headgroups, glycerol and carbonyl groups, interfacial water, counterions, and hydrocarbon chains. Therefore, for membrane-active peptides and proteins, partitioning is commonly described using operational definitions that specify how the membrane-associated population is quantified.

In vesicle-based assays, the membrane-associated population can be estimated by direct separation methods, such as centrifugation, ultracentrifugation, sedimentation, or flotation, or indirectly by titrating lipid vesicles into a fixed concentration of peptide or protein and monitoring a membrane-sensitive observable. At sufficiently low peptide-to-lipid ratios, and when only the accessible leaflet is considered, partitioning may be expressed in mole-fraction form as ${K}_{\mathrm{x}}=({\left[P\right]}_{\mathrm{m}\mathrm{e}\mathrm{m}\mathrm{b}}/{\left[L\right]}_{\mathrm{a}\mathrm{c}\mathrm{c}})/({\left[P\right]}_{\mathrm{a}\mathrm{q}}/\left[W\right])$, where ${\left[P\right]}_{\mathrm{m}\mathrm{e}\mathrm{m}\mathrm{b}}$ and ${\left[P\right]}_{\mathrm{a}\mathrm{q}}$ are the membrane-associated and aqueous concentrations of the molecule, ${\left[L\right]}_{\mathrm{a}\mathrm{c}\mathrm{c}}$ is the lipid concentration effectively accessible from the aqueous phase, and $\left[W\right]$ is the molar concentration of water (~ 55.5 M) (Sadi et al. 2023). The corresponding free energy of partitioning can be written as Δ ${G}_{\mathrm{p}\mathrm{a}\mathrm{r}\mathrm{t}}=-RTln {K}_{\mathrm{x}}$, providing a thermodynamic measure of the transfer from water to the membrane-associated state.

In titration experiments, the membrane-associated fraction can be related to an experimental observable according to ${Y}_{\mathrm{o}\mathrm{b}\mathrm{s}}={Y}_{\mathrm{a}\mathrm{q}}+({Y}_{\mathrm{m}\mathrm{e}\mathrm{m}}-{Y}_{\mathrm{a}\mathrm{q}}){f}_{\mathrm{b}}$, where ${Y}_{\mathrm{o}\mathrm{b}\mathrm{s}}$ is the measured signal, ${Y}_{\mathrm{a}\mathrm{q}}$ and ${Y}_{\mathrm{m}\mathrm{e}\mathrm{m}}$ are the limiting signals of the aqueous and membrane-associated states, and ${f}_{\mathrm{b}}$ is the membrane-associated fraction. When lipid concentration is much higher than peptide or protein concentration, this fraction can be approximated as ${f}_{\mathrm{b}}={K}_{\mathrm{L}}{[L]}_{\mathrm{a}\mathrm{c}\mathrm{c}}/(1+{K}_{\mathrm{L}}{[L]}_{\mathrm{a}\mathrm{c}\mathrm{c}})$, where ${K}_{\mathrm{L}}={K}_{\mathrm{x}}/[W]$. A rigorously defined partition coefficient is a method-independent equilibrium quantity. In practice, however, membrane association is often coupled to folding, insertion, oligomerization, changes in local mobility, dehydration, and lipid reorganization. As a result, spectroscopic and calorimetric titrations may yield apparent or operational association parameters when the monitored observable is selectively sensitive to particular membrane-associated states. Fluorescence may report changes in local polarity or insertion depth, circular dichroism may preferentially report the membrane-bound folded population, electron spin resonance may distinguish mobile and motionally restricted components, and calorimetry reports the global heat of coupled binding and membrane reorganization processes. Thus, partition coefficients and apparent membrane-association parameters provide a quantitative starting point for membranotropism, but they must be interpreted in relation to the molecular state and experimental model used to define the membrane-associated population.

For cationic peptides, the initial stage of membrane association is often dominated by electrostatic surface enrichment. Negatively charged membranes can concentrate positively charged peptides near the bilayer surface, increasing their local concentration by orders of magnitude relative to the bulk aqueous phase. In such cases, apparent partition coefficients may overestimate the intrinsic hydrophobic tendency of a molecule to enter the membrane if electrostatic contributions are not explicitly considered. Studies with magainin 2 amide and nisin Z illustrate this principle: association with anionic membranes can display strong apparent affinity and exothermic heats of binding, but these values reflect a combination of electrostatic attraction, interfacial partitioning, peptide redistribution, and membrane perturbation rather than a single molecular force (Wenk and Seelig 1998; Breukink et al. 2000).

After surface enrichment, membrane-active molecules may partition into the bilayer interface, where amphipathic structures can maintain polar contacts while burying hydrophobic and aromatic groups more deeply. The interfacial hydrophobicity scale developed by Wimley and White showed that transfer from water to the membrane interface follows an energetic logic distinct from transfer into a hydrocarbon-like core: aromatic residues are particularly favored at the interface, whereas charged residues and peptide bonds are energetically costly when deeply buried (Wimley and White 1996; White and Wimley 1999). Thus, interfacial partitioning can stabilize membrane-associated states without requiring complete insertion across the bilayer (Jacobs and White 1989).

Progression from interfacial binding to deeper insertion requires additional energetic compensation. Favorable hydrophobic contacts are opposed by dehydration, conformational restriction, lipid displacement, loss of translational and rotational freedom, and deformation of the bilayer (Jacobs and White 1989; Wimley and White 1996; Nielsen et al. 1998). Hydrophobic mismatch exemplifies this balance: when the hydrophobic length of an inserted peptide or protein differs from the bilayer thickness, surrounding lipids must deform, generating an elastic cost that influences stability and function. In gramicidin A channels, for instance, changes in bilayer thickness alter channel lifetime, showing that membrane insertion depends not only on peptide structure but also on the mechanical properties of the lipid matrix (Elliott et al. 1983). Andersen and Koeppe later generalized this behavior into a broader energetic description, in which hydrophobic mismatch is understood as a coupling between protein insertion and bilayer deformation, with the associated energetic cost depending on bilayer thickness, intrinsic curvature, and elastic moduli (Nielsen et al. 1998; Andersen and Koeppe 2007).

Beyond equilibrium partitioning and insertion depth, membrane association can also be limited by kinetic and elastic energy barriers along the pathway toward deeper insertion or membrane remodeling. Transfer of a peptide or protein segment from water to the bilayer interface or core requires dehydration, lipid displacement, and local membrane deformation, whereas fusion and pore formation require the membrane to pass through highly curved nanometer-scale intermediates. These barriers are therefore not determined only by the molecular structure of the peptide or protein, but also by membrane tension, curvature, lateral pressure, thickness, and elastic moduli (Chernomordik and Kozlov 2003; Kozlov and Chernomordik 2015). In a simplified description, membrane tension contributes an additional area-work term to the free-energy barrier, which can be approximated as Δ ${G}_{\upgamma }\approx \gamma \Delta A$, where γ is the membrane tension and Δ A is the effective membrane area change associated with the intermediate state. Thus, even modest changes in membrane tension can substantially alter the energetic cost of insertion, pore formation, or fusion when these processes require membrane expansion or highly curved intermediates. Recent quantitative estimates for virus–endosome fusion illustrate the magnitude of this effect. Chow et al. estimated that the bending/elastic work required to reach a pore-competent state is approximately 43–65 *k*_B_*T*, whereas the residual energetic margin provided by three influenza HA trimers after this pore-bending cost is approximately 37–59 *k*_B_*T* (Chow et al. 2026). Under swollen endosomal conditions, an increase in membrane tension to approximately 5 mN/m would add an area-work penalty of about 60 *k*_B_*T* for Δ A = 50 nm^2^, or about 120 *k*_B_*T* for Δ A = 100 nm^2^, potentially exhausting the energetic budget available for fusion-pore formation (Chow et al. 2026). Thus, membrane mechanics can determine whether the energy released by protein refolding, insertion, or oligomerization is sufficient to drive productive membrane remodeling.

Membrane binding is also frequently coupled to conformational transitions. Amphipathic peptides that are disordered in solution may fold into α-helices at the membrane interface, improving the segregation of polar and nonpolar residues. This folding can contribute favorably to the observed binding enthalpy by stabilizing intramolecular hydrogen bonds and optimizing peptide–lipid interactions, but it also imposes an entropic penalty by restricting conformational freedom. Therefore, a strongly exothermic calorimetric signal should not be interpreted directly as proportionally strong membrane affinity (Wieprecht et al. 1999). On the other hand, β-structure formation often involves both membrane binding and intermolecular peptide–peptide association. In these cases, the membrane acts not only as a binding surface but also as a two-dimensional concentrating and orienting platform that can promote β-sheet extension, aggregation, pore formation, or amyloid-like assemblies (Meier and Seelig 2008). At higher surface densities, simple partition equilibria often break down, and membrane-active peptides may transition into thinning, defect-forming, or pore-forming states (Wenk and Seelig 1998; Huang 2000). The thermodynamic description must therefore follow the specific molecular state being populated, whether adsorption, folding, insertion, oligomerization, leakage, or pore formation.

Overall, the thermodynamic determinants of membranotropism reflect the balance between favorable and unfavorable contributions. Favorable terms may include electrostatic attraction, counterion release, hydrogen bonding, van der Waals contacts, hydrophobic partitioning, release of structured water, and stabilization of folded or oligomeric states. Opposing terms may include dehydration of polar groups, loss of translational, rotational, or conformational entropy, lipid ordering, membrane deformation, and the energetic cost of creating defects or pores. The same molecule may therefore appear entropy-driven, enthalpy-driven, or electrostatically amplified depending on the membrane composition, ionic strength, concentration regime, and experimental model. This complexity reinforces the view that membranotropism is a coupled thermodynamic process emerging from reciprocal adaptation between molecular architecture and membrane biophysics.

## Experimental and computational approaches to investigate membranotropism

Because membranotropism involves a continuum of membrane-associated states, no single technique can fully characterize it. Surface adsorption, interfacial partitioning, insertion, membrane-induced folding, oligomerization, pore formation, translocation, curvature generation, and membrane remodeling occur over different length and time scales and are therefore best investigated by combining complementary experimental and computational approaches (Galdiero et al. 2013; Clifton et al. 2020; Ulmschneider and Ulmschneider 2018).

A first level of analysis is the quantitative evaluation of membrane binding and partitioning. Equilibrium lipid titrations monitored by intrinsic tryptophan fluorescence, extrinsic fluorophores, fluorescence anisotropy, Förster resonance energy transfer (FRET), circular dichroism (CD), electron spin resonance (ESR), nuclear magnetic resonance (NMR), or other environment-sensitive observables can provide membrane-bound fractions and apparent partition coefficients when the signal reports on the membrane-associated state (White and Wimley 1999; Ladokhin et al. 2000). These approaches are particularly useful for comparing lipid selectivity, charge dependence, sterol effects, or peptide-to-lipid ratio dependence, but the resulting parameters must be interpreted according to the molecular state probed by each observable. Isothermal titration calorimetry (ITC) provides a complementary label-free thermodynamic readout of membrane association, including apparent affinity, enthalpy, entropy, and stoichiometry, whereas surface plasmon resonance (SPR) and related surface-sensitive methods can resolve association and dissociation kinetics at membrane-mimetic interfaces (Seelig 1997; Beseničar et al. 2006; Clifton et al. 2020).

Spectroscopic methods probe the structural and dynamic transitions that accompany membrane association. CD reports membrane-induced secondary-structure formation, whereas oriented CD can provide information on peptide alignment relative to macroscopically oriented lipid bilayers (Bürck et al. 2016). Fourier-transform infrared spectroscopy (FTIR), including attenuated total reflection FTIR, is also widely used to analyze peptide and protein secondary structure, orientation, hydration, and lipid-associated conformational changes in membrane environments (Tamm and Tatulian 1997). Steady-state and time-resolved fluorescence spectroscopy can monitor changes in local polarity, hydration, quenching accessibility, rotational mobility, membrane leakage, lipid mixing, and peptide orientation or insertion depth when suitable probes are available (Stryer and Haugland 1967; Ladokhin et al. 2000; Eftink and Ghiron 1981).

Magnetic resonance methods provide complementary information on structure, topology, and dynamics. Solution NMR can characterize peptides and small proteins in detergent micelles, bicelles, or other membrane-mimetic environments, whereas solid-state NMR enables structural and dynamic studies in lipid bilayers, oriented membranes, and proteoliposomes, including systems that more closely preserve the anisotropic properties of membranes (Opella 2015; Opella and Marassi 2017). ESR spectroscopy is particularly powerful because it can report on both sides of lipid–protein interactions: spin-labeled lipids provide information on membrane order, fluidity, phase behavior, and perturbation, whereas site-directed spin labeling of proteins or peptides can reveal topology, insertion depth, mobility, conformational changes, and intermolecular distances (Schreier et al. 1978; Marsh 2008a; Basso et al. 2016b, a). Together, NMR and ESR are especially useful for distinguishing surface-bound, interfacially inserted, and more deeply embedded states.

Calorimetric methods connect membrane association with thermodynamic and cooperative transitions. ITC reports heat changes associated with adsorption, partitioning, folding, insertion, aggregation, or lipid reorganization during titration experiments. Differential scanning calorimetry (DSC), in turn, monitors lipid phase transitions and, when applicable, protein thermal unfolding or melting, allowing one to evaluate how peptides or proteins alter lipid packing, cooperativity, transition temperature, and enthalpy, as well as how the membrane environment stabilizes or destabilizes membrane-associated proteins (McElhaney 1986; Micheletto et al. 2017). These methods are particularly informative when membrane association is coupled to changes in lipid order, phase coexistence, or protein stability.

Scattering, imaging, and microscopy-based approaches extend this information to supramolecular and mesoscale organization. Small-angle X-ray and neutron scattering can quantify bilayer thickness, lamellar organization, peptide-induced structural changes, and membrane remodeling in solution (Nielsen et al. 2023). X-ray crystallography and cryo-EM provide high-resolution structural information on membrane proteins and larger complexes stabilized in detergents, lipidic cubic phases, amphipols, nanodiscs, or other membrane-mimetic platforms, although the choice of mimetic can strongly influence the observed conformation, oligomeric state, and lipid interactions (Kermani 2021; Choy et al. 2021; Sun and Gennis 2019). Cryo-EM, cryo-electron tomography, and cryo-TEM can also directly visualize membrane deformation, vesicle disruption, pore-like defects, or peptide-induced restructuring in vitrified samples, thereby preserving hydrated membrane systems in near-native states and enabling morphological analysis of peptide-induced membrane damage (Dubochet et al. 1988; Han et al. 2009; Chen et al. 2023). Atomic force microscopy (AFM) and high-speed AFM provide nanoscale topographic, mechanical, and dynamic information on supported membranes and membrane-associated assemblies, including membrane remodeling and protein activity at interfaces (Clifton et al. 2020).

Giant unilamellar vesicles (GUVs) combined with confocal or other optical microscopy are especially useful for visualizing membrane domains, phase separation, curvature generation, budding, tubulation, leakage, fusion, and condensate–membrane coupling in cell-sized model membranes (Riske and Dimova 2005; Dimova 2019). In parallel, functional assays such as vesicle leakage, dye influx, content mixing, lipid mixing, electrophysiological measurements, and cell-based membrane integrity assays are essential for connecting molecular association with membrane permeability, fusion, pore formation, or translocation.

Molecular dynamics simulations provide a complementary molecular view of adsorption, reorientation, insertion, oligomerization, pore formation, translocation, and lipid reorganization. Atomistic simulations can describe residue-level contacts, hydration, lipid specificity, and local bilayer deformation, whereas coarse-grained and enhanced-sampling approaches extend the accessible length and time scales and allow exploration of cooperative or rare events such as peptide clustering, membrane permeation, and pore nucleation (Ulmschneider and Ulmschneider 2018). However, simulations are most informative when benchmarked against experimental observables, since force-field accuracy, sampling limitations, membrane composition, and finite system size can strongly affect the inferred mechanism. For this reason, robust characterization of membranotropism usually requires integrated strategies that combine quantitative binding or partitioning measurements with structural, spectroscopic, thermodynamic, imaging, functional, and computational readouts.

## Architectures and interaction modes of membranotropic proteins and peptides

Protein and peptide interactions with lipid membranes underlie diverse biological processes, including antimicrobial defense, membrane fusion, signaling, transport, membrane remodeling, and toxin activity. These interactions are controlled not only by local structural motifs, but also by the broader architecture of the membrane-active molecule (Tubiana et al. 2022; White and Wimley 1999; Wimley and White 1996; Ladokhin and White 1999; Wimley 2010; Marsh 2008b). Here, “architecture” refers to the spatial organization, topology, conformational accessibility, and cooperative assembly of membrane-interacting elements within peptides, proteins, or supramolecular systems. This perspective helps explain why short amphipathic peptides, β-structured toxins, amphitropic proteins, lipidated signaling proteins, monotopic enzymes, and oligomeric pore-forming assemblies can engage membranes through distinct mechanisms and produce different biological outcomes (Hong and Su 2011; Kabelka and Vácha 2021; White and Wimley 1999).

Rather than defining rigid structural classes, molecular architecture determines how a system samples membrane-associated states and converts membrane recognition into function (Lee 2004). The outcome of this process depends on the interplay between molecular features, such as charge distribution, hydrophobicity, amphipathicity, conformational flexibility, lipid-binding domains, and oligomerization propensity, and membrane properties, including lipid composition, charge, packing, curvature, thickness, cholesterol content, and phase state. This architectural perspective organizes the major classes discussed below: short membranotropic peptides and toxins, peripheral and amphitropic proteins, secreted and extracellular membrane modulators, and oligomeric assemblies involved in pore formation and membrane remodeling.

### Short membranotropic peptides

Short membranotropic peptides, including antimicrobial peptides (AMPs), peptide toxins, viral fusion segments, anticancer peptides, cell-penetrating peptides, and amyloidogenic peptides, represent some of the most versatile membrane-active architectures. Many of these molecules are relatively small, conformationally adaptable, and enriched in residues that promote electrostatic attraction, interfacial anchoring, amphipathic folding, aromatic stabilization, or cooperative assembly (Hancock and Sahl 2006; Wimley 2010; Zasloff 2002; Anderluh and Macek 2002; King 2011). Their activity often emerges from the coupling between membrane binding and structural rearrangement: peptides that are partially disordered in aqueous solution may acquire α-helical, β-hairpin, β-sheet, or extended membrane-bound conformations upon interaction with lipid bilayers. This disorder-to-order transition can optimize hydrophobic burial, electrostatic interactions, hydrogen bonding, and interfacial packing, thereby linking peptide architecture to membrane affinity and function (White and Wimley 1999; Wimley 2010).

**Fig. 1:**
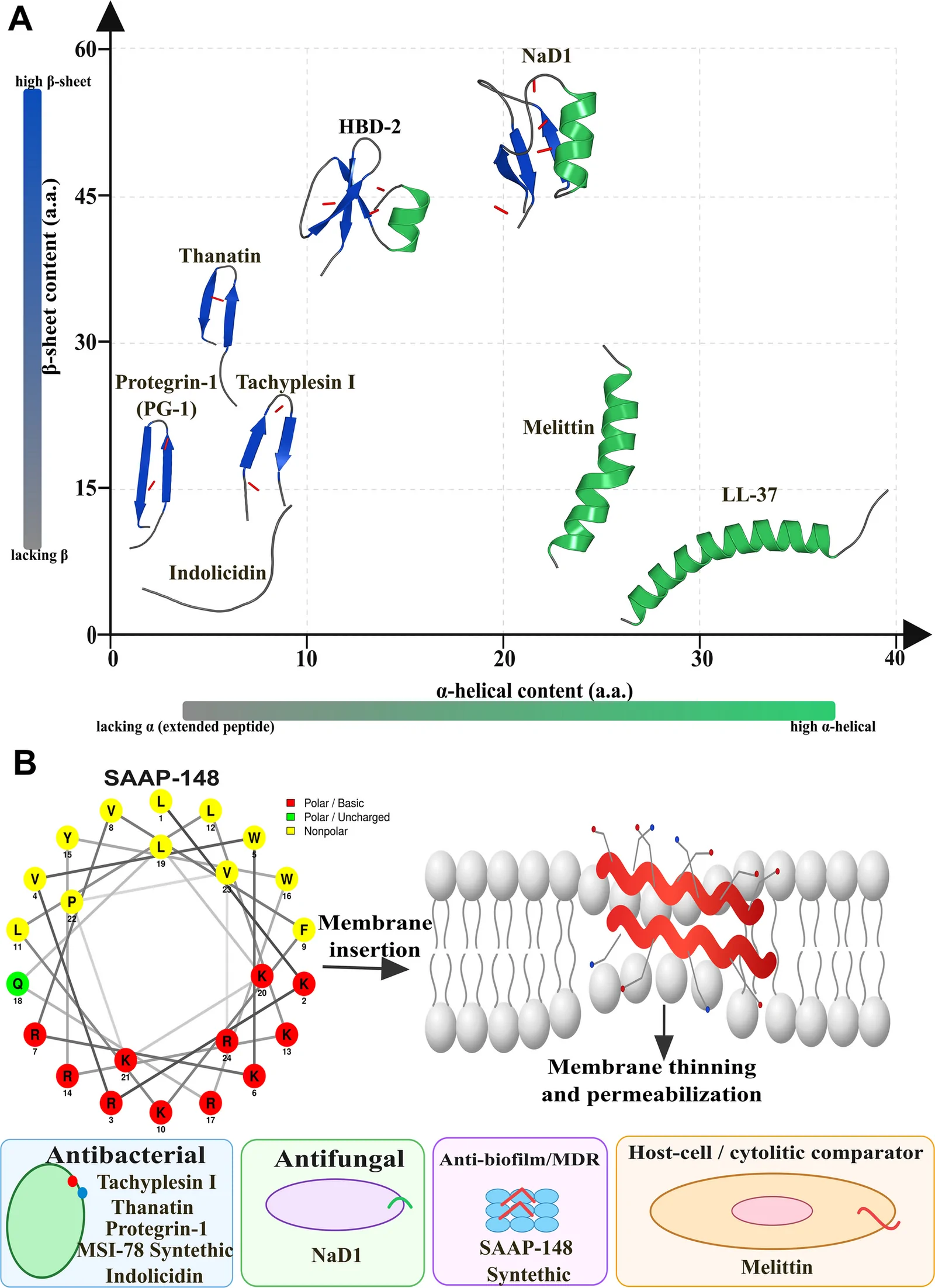
Structural diversity and membranotropic behavior of representative antimicrobial peptides. **A** Structural distribution of AMPs according to their α-helical and β-sheet content, including peptides from human host defense systems, invertebrates, plants, and venom-derived sources. α-helices are shown in green, β-strands in blue, and disulfide bonds in red. Protein Data Bank IDs: 2K6O, 1ZY6, 2RTV, 8GAJ, 6CS9, 1MR4, 1G89, and 8AHT. **B** Functional and mechanistic representation of membranotropic AMPs. SAAP-148 is highlighted as a rationally designed LL-37-derived peptide with enhanced cationicity and α-helical amphipathicity achieved through substitutions with cationic residues. These features promote membrane insertion, bilayer thinning, and permeabilization, illustrating how rational design can transform a natural host-defense peptide into a synthetic candidate with enhanced antibacterial, antibiofilm, and anti-MDR activity. Additional examples include AMPs with antibacterial activity (tachyplesin I, thanatin, protegrin-1, MSI-78), antifungal activity (NaD1), antibiofilm/MDR-oriented activity (SAAP-148), and cytolytic comparator activity (melittin)

AMPs are paradigmatic examples of this principle because their structural diversity is closely associated with functional versatility (Hancock and Sahl 2006; Zasloff 2002). These peptides operate at the interface between host defense, membrane biophysics, and microbial adaptation, combining direct antimicrobial activity with immunomodulatory, antibiofilm, antifungal, anticancer, and, in some cases, cytolytic functions (Mookherjee et al. 2020). As shown in Fig. 1A, representative AMPs occupy distinct regions of structural space according to their α-helical and β-sheet content. α-Helical peptides such as LL-37, magainin-derived peptides, and melittin display amphipathic architectures that favor interfacial partitioning and lipid-packing perturbation (Bechinger 1997; Henzler-Wildman et al. 2004; Matsuzaki et al. 1997), whereas β-sheet or β-hairpin peptides such as protegrin-1, tachyplesin I, thanatin, and defensins often rely on compact folds, disulfide stabilization, electrostatic membrane recognition, and cooperative permeabilization (Huynh et al. 2023; Kushibiki et al. 2014). Mixed α/β architectures are represented by defensins such as human β-defensin 2 (Järvå et al. 2018) and the plant defensin NaD1 (Lay et al. 2003), whereas peptides such as indolicidin adopt extended conformations lacking canonical secondary structure (Rozek et al. 2000). Together, these examples show that AMP membranotropism does not rely on a single fold but emerges from the integration of charge, amphipathicity, hydrophobicity, aromatic residues, conformational plasticity, and membrane context (Hancock and Sahl 2006; Wimley 2010).

This functional diversity extends from natural peptides to rationally designed or chemically modified analogues optimized for improved stability, selectivity, and activity under physiological conditions (Bellotti and Remelli 2022; de Breij et al. 2018). As illustrated in Fig. 1B, SAAP-148 provides a representative example of structure-guided AMP design. Derived from the functional C-terminal region of LL-37, this peptide was engineered to preserve α-helical amphipathicity while increasing net positive charge through substitutions with cationic residues such as arginine and lysine (de Breij et al. 2018). These modifications enhanced cationicity, amphipathic character, stability, and biological activity, favoring rapid interaction with bacterial membranes, perturbation of the bilayer hydrophobic core, membrane thinning, and permeabilization. Similar principles are illustrated by other experimental systems (de Breij et al. 2018). Synthetic Pantinin peptides derived from scorpion venom acquire α-helical structure upon membrane binding, preferentially interact with anionic model membranes, and display selective antitumor activity, while cholesterol reduces peptide binding and membrane permeabilization, highlighting how charge and sterol content can modulate peptide selectivity toward cancer-like membranes (Crusca et al. 2018). In Ctx(Ile21)-Ha and paramagnetic analogues, lipid composition modulates peptide topology, dynamics, and membrane-disruptive activity: PE-containing bacterial-like membranes favor α-helical coiled-coil structures, whereas SM-containing mammalian-like membranes support different interaction modes, emphasizing how subtle sequence modifications and membrane composition jointly control AMP function (Vicente et al. 2022). More recently, the bioinspired peptide CaDef2.1G27-K44 (CDF-GK) combined antifungal and antibiofilm activity against *Candida krusei* with membrane permeabilization, ergosterol interaction, intracellular effects, and low toxicity in a *Galleria mellonella* infection model, illustrating how membranotropic design principles can be linked to translational antifungal strategies (Guimarães et al. 2025).

Peptide toxins and other short membrane-active segments follow related principles, although they may display higher target specificity through receptor-assisted recognition, lipid selectivity, or defined oligomerization pathways. Actinoporins, for example, recognize sphingomyelin-containing membranes before undergoing oligomerization and pore formation, whereas other peptide toxins combine interfacial partitioning with specific lipid or protein recognition (Anderluh and Macek 2002; King 2011). Amyloidogenic and intrinsically disordered membrane-active peptides provide additional examples in which membrane binding, local concentration, β-structure formation, and aggregation can be coupled to membrane permeabilization or nanoscale defect formation (Uversky 2019). These systems emphasize that short peptides can use membrane surfaces not only as targets, but also as organizing platforms that promote folding, clustering, and functional assembly.

Viral membrane-active peptides constitute another major class of short membranotropic elements. They use diverse biophysical strategies to interact with host membranes, including fusion, permeabilization, ion-channel formation, and translocation (Falanga et al. 2018; Harrison 2015). Viral fusion peptides are typically hydrophobic or amphipathic segments of larger fusion proteins that become exposed or repositioned during viral entry and insert into the target membrane. Because membrane fusion requires the apposition of two bilayers, dehydration of opposing membrane interfaces, lipid rearrangement, stalk formation, hemifusion, and fusion-pore opening, this process involves substantial kinetic and elastic energy barriers that must be overcome by coordinated protein refolding and membrane remodeling (Chernomordik and Kozlov 2003; Jahn et al. 2003; White et al. 2008). Importantly, these barriers are not fixed properties of the viral protein alone. They also depend on the mechanical state of the target membrane, including membrane tension, curvature, hydration, lipid packing, and the energetic cost of generating highly curved fusion intermediates.

Class I fusion-derived peptides, such as those found in influenza HA2, HIV-1 gp41, coronavirus Spike S2, and Ebola GP2, participate in conserved refolding pathways involving pre-hairpin intermediates and six-helix bundle formation, thereby coupling fusion peptide insertion to large-scale membrane merger (White et al. 2008). Studies with SARS-CoV and SARS-CoV-2 Spike fusion peptides further highlight the importance of membrane physicochemistry in this process. These peptides can increase lipid packing and acyl-chain ordering, reduce interfacial hydration, promote membrane permeabilization, and reorganize lipid bilayers in a sequence-dependent manner, with complete internal fusion peptide segments displaying stronger fusogenic and membrane-remodeling activity than shorter fusogenic regions (Basso et al. 2016b, a; 2021). Together, these findings support the view that viral fusion depends not only on conformational changes in viral proteins, but also on peptide-induced alterations in lipid packing, hydration, permeability, and curvature stress.

At the cellular level, the efficiency of viral fusion also depends on whether the target membrane provides a mechanically permissive environment for progression through hemifusion and fusion-pore formation. A recent study on PIKfyve-dependent viral entry provides a direct example of this principle. Inhibition of the lipid kinase PIKfyve by apilimod, or transient hypotonic treatment, induced swelling of late endosomes and lysosomes and selectively blocked entry of enveloped viruses that depend on late endosomal fusion, including virions bearing Ebola virus GP or SARS-CoV-2 Spike (Chow et al. 2026). This effect was attributed primarily to increased endosomal membrane tension rather than to defective trafficking, loss of endosomal acidity, or phosphoinositide depletion alone. The authors further framed fusion-pore formation as an energetic competition in which the elastic work required to generate highly curved pore intermediates can be increased by membrane tension through an area-work term, ΔG_γ_ ≈ γΔA. Thus, viral membranotropism depends not only on conformational changes in viral proteins and exposure of fusion peptides, but also on the mechanical state of the target membrane and its capacity to support the lipid rearrangements required for hemifusion, fusion-pore expansion, and genome release.

Class II fusion systems, such as flavivirus E proteins, rely on a different architecture, in which hydrophobic fusion loops insert into the membrane interface following pH-triggered conformational rearrangements (Falanga et al. 2018; Modis et al. 2004). Beyond canonical fusion peptides, viruses also encode nonclassical membrane-active elements. Some viral peptides act as membrane-disruptive sequences that destabilize lipid bilayers and promote permeabilization or genome release, whereas viroporins form small oligomeric channels that modify ion homeostasis and intracellular organelle environments to support replication, assembly, or viral release (Nieva et al. 2012). Arginine-rich cell-penetrating peptides, such as HIV-1 Tat-derived sequences, exploit electrostatic interactions with negatively charged membranes and may facilitate translocation through transient thinning, poration, or endocytic pathways (Mishra et al. 2011). Thus, viral membranotropism is not limited to fusion but includes a broader repertoire of membrane-associated mechanisms.

The functional outcomes of short membranotropic peptides are therefore highly diverse. Depending on sequence, concentration, lipid composition, membrane charge, curvature, and packing, these peptides may induce transient defects, membrane thinning, curvature stress, pore formation, membrane fusion, intracellular translocation, or non-lytic remodeling (Wimley 2010). Thus, short membranotropic peptides should not be regarded simply as membrane-disrupting agents, but as adaptable molecular architectures capable of sampling multiple membrane-bound states and translating local membrane properties into biological function.

### Peripheral, integral, monotopic, and amphitropic proteins

Beyond short peptides, larger proteins expand this continuum through regulated membrane-binding domains, lipid anchors, partial insertion motifs, and transmembrane architectures. The traditional distinction between peripheral and integral membrane proteins is useful but incomplete, since it does not fully capture the structural and dynamic complexity observed in living systems. Integral membrane proteins contain transmembrane α-helical (von Heijne 2006) or β-barrel (Schulz 2002) segments that are deeply embedded in the bilayer, whereas peripheral membrane proteins associate more transiently with membrane surfaces through electrostatic interactions, lipid-binding domains, shallow hydrophobic insertion, or protein–protein interactions (Lemmon 2008; McLaughlin and Murray 2005). However, many membrane-associated proteins do not fit neatly into either category. Membrane association is better understood as a dynamic continuum defined by insertion depth, residence time at the membrane interface, reversibility of binding, conformational adaptability, and dependence on local lipid composition (Cho and Stahelin 2005; Ladokhin and White 1999; Lemmon 2008; White and Wimley 1999).

This continuum is particularly evident in proteins involved in signaling and membrane organization, in which reversible membrane recruitment controls spatial localization, enzymatic activity, supramolecular assembly, curvature generation, and mechanical coupling. Representative examples are summarized in Table 1. Lipid-sensing proteins such as PLCδ1 and AKT1 use PH domains to recognize phosphoinositide signatures and recruit signaling enzymes to defined membrane compartments (Ferguson et al. 1995; Milburn et al. 2003). Membrane-remodeling proteins such as epsin and amphiphysin couple lipid recognition to amphipathic helix insertion or BAR-domain scaffolding (Boucrot et al. 2012; Peter et al. 2004), whereas force-coupled systems such as integrins and talin integrate membrane-associated conformational changes with cytoskeletal tension (Del Rio et al. 2009; Zhu et al. 2008). Caveolin-1 further illustrates how cholesterol-dependent oligomerization can organize membrane platforms (Liebl and Voth 2024). These examples show that membranotropism can regulate signaling and membrane organization without necessarily causing membrane disruption.

**Table 1:** Representative classes of membranotropic proteins involved in signaling and membrane organization

Protein	Function or cell signaling	Biophysical mechanism	Ref.
I. Lipid-sensing membranotropism (phosphoinositide recognition)			
Phospholipase C delta-1 (PLCδ1)	PIP_2_ hydrolysis coupled to Ca^2+^ signaling via IP_3_/DAG production	High-affinity PIP_2_ binding via pleckstrin homology (PH) domain (Lys/Arg-rich pocket); non-disruptive electrostatic membrane targeting	Ferguson et al. (1995)
AKT1 (Protein kinase B, PKB)	PI3K-dependent signaling controlling cell survival and metabolism	Pleckstrin homology (PH) domain binds PIP_3_ enabling membrane recruitment and conformational activation of the kinase	Milburn et al. (2003)
II. Membrane-remodeling membranotropism (insertion and curvature generation)			
Epsin 1 (ENTH domain)	Clathrin mediated endocytosis and coated-pit formation	PIP_2_-triggered amphipathic helix insertion induces membrane curvature	Boucrot et al. (2012)
Amphiphysin (BAR domain)	Endocytosis and membrane tubulation during vesicle formation	Crescent-shaped BAR dimer scaffolds negatively charged membranes and senses/induces curvature	Peter et al. (2004)
III. Insertion-driven membranotropism (amphipathic helix)			
ADP-ribosylation factor 1 (ARF1)	Vesicle trafficking (Golgi), COPI coat recruitment	GTP-dependent exposure of N-terminal myristoylated amphipathic helix inserts into membranes, driving curvature and coat assembly	Carlos Amor et al. (1994)
IV. Lipid-clustering/nanodomain membranotropism			
K-Ras4B	MAPK signaling	Lipid-dependent nanocluster formation via electrostatic interactions with anionic lipids and lipid sorting	Sun et al. (2007)
MARCKS	PIP_2_ regulation	Electrostatic sequestration and clustering of PIP_2_ through polybasic effector domain	Gambhir et al. (2004)
V. Mechanotransductive membranotropism (force-coupled systems)			
Integrin alpha IIb beta 3 (αIIbβ3)	Cell adhesion and outside-in signaling	Force-induced conformational activation; ligand binding couples to membrane tension and cytoskeletal forces	Zhu et al. (2008)
Talin 1	Integrin activation and focal adhesion assembly	Force-dependent unfolding of talin rod exposes vinculin-binding sites; couples integrins to actin and transduces mechanical tension	Del Rio et al. (2009)
VI. Cholesterol-dependent platform organization			
Caveolin-1 (CAV1)	Caveolae formation and membrane organization	Cholesterol-dependent oligomerization drives membrane domain formation	Liebl and Voth (2024)

Membrane-remodeling proteins illustrate how peripheral or partially inserted architectures can convert membrane recognition into changes in bilayer morphology. Although lipid composition, leaflet asymmetry, and curvature stress contribute to membrane deformation, efficient generation, and stabilization of highly curved structures often require membrane-associated proteins that lower the energetic cost of bending (McMahon and Gallop 2005). These proteins can remodel membranes through nonexclusive mechanisms, including curved scaffolds, shallow insertion of amphipathic helices, local changes in spontaneous curvature, protein crowding, and bilayer-couple effects (Jarsch et al. 2016).

BAR-domain proteins provide a representative example of this coupling between membrane recognition and curvature remodeling. Their curved, positively charged surfaces interact preferentially with anionic membranes and can stabilize or generate curved regions during endocytosis and vesicular trafficking (Frost et al. 2009; Gallop et al. 2006; Peter et al. 2004). However, BAR-domain activity is not explained solely by a simple scaffold model. Amphipathic helix insertion, lipid-packing defects, membrane tension, protein density, and oligomerization also contribute to curvature sensing and generation (Gallop et al. 2006; Simunovic et al. 2016). Thus, BAR proteins exemplify a broader principle: membrane remodeling emerges from cooperation between protein architecture, membrane mechanics, and local protein concentration (Frost et al. 2009; Simunovic et al. 2016).

Amphitropic proteins provide some of the clearest examples of this dynamic continuum. These proteins reversibly cycle between cytosolic and membrane-associated states in response to regulatory signals, using transient membrane engagement as a mechanism for spatial and temporal control (Burn 1988; Johnson and Cornell 1999; Murray et al. 1999). Their membrane association is frequently governed by the cooperative action of lipid anchors, clusters of basic residues, phosphorylation sites, calcium-sensitive conformational switches, and cryptic amphipathic segments. In c-Src family kinases, membrane recruitment depends on cooperation between an N-terminal myristoyl group and adjacent basic residues, integrating hydrophobic insertion with electrostatic interactions with anionic phospholipids (Buser et al. 1994; Sigal et al. 1994). MARCKS proteins operate through a related but reversible electrostatic mechanism, in which a polybasic effector domain binds phosphatidylserine-rich membranes and laterally sequesters PI(4,5)P_2_; phosphorylation weakens this association and promotes cytosolic release (Glaser et al. 1996; Kim et al. 1994). Recoverin and other calcium-myristoyl switch proteins illustrate a third strategy, in which Ca^2+^ binding exposes an otherwise buried myristoyl group, allowing reversible membrane insertion (Dizhoor et al. 1993; Zozulya and Stryer 1992).

Septins further expand this amphitropic repertoire by associating with phosphoinositide-rich membranes through specialized membrane-binding motifs and amphipathic elements that support curvature sensing, scaffold formation, and higher-order filament assembly (Mostowy and Cossart 2012; Cannon et al. 2019). Unlike classical BAR-domain proteins, which often sense or impose curvature at the nanometer scale, septins can display sensitivity to curvature at the micrometer scale and can also induce membrane tubulation or deformation through filament assembly (Beber et al. 2019; Nakazawa et al. 2023). Studies on *Schistosoma mansoni* septins provide a useful example of how specific membrane-interacting regions control septin–lipid association: SmSEPT10 interacts directly with lipid membranes through its C-terminal domain and forms organized complexes at vesicle surfaces, whereas subsequent work identified specific lipid-interacting motifs within this region (Cavini et al. 2024; Zeraik et al. 2016). These findings position septins as amphitropic membrane-associated scaffolds that couple lipid recognition, filament assembly, curvature sensing, and membrane remodeling.

Bacterial lipid-biosynthetic enzymes provide additional examples of how membrane association regulates catalysis and substrate channeling. PlsX, the first enzyme of the phosphatidic acid pathway in Gram-positive bacteria, behaves as a peripheral membrane enzyme whose association with anionic phospholipids is mediated by electrostatic interactions and by a hydrophobic loop located at the tip of amphipathic dimerization helices (Sastre et al. 2020a, 2020b). Importantly, PlsX membrane binding and insertion can be functionally separated: charge favors membrane association, whereas fatty-acid saturation and bilayer fluidity modulate the insertion step, which is required for efficient acyl-phosphate delivery to the integral membrane partner PlsY rather than for soluble catalytic activity itself (Sastre et al. 2020a, 2020b). PatA, an essential mycobacterial acyltransferase involved in phosphatidyl-myo-inositol mannoside biosynthesis, illustrates a related but more deeply embedded interfacial strategy. This enzyme is tightly anchored to anionic lipid bilayers through a two-helix structural motif and electrostatic interactions, positioning its active site at the membrane–water interface, where it can couple recognition of membrane-embedded glycolipid substrates with soluble acyl-CoA donors through a hydrocarbon ruler mechanism (Anso et al. 2021). Together, PlsX and PatA show that membrane association in lipid-metabolic enzymes is not merely a localization mechanism, but a functional determinant of substrate access, catalytic coupling, and pathway organization.

Monotopic proteins occupy an intermediate architectural state. Rather than spanning the entire bilayer, they insert deeply into only one leaflet, allowing stable membrane association while preserving asymmetric interaction with the membrane interface. Cyclooxygenases and prostaglandin synthases illustrate this mode of binding (Allen et al. 2019; Picot et al. 1994). Such proteins show that membrane engagement cannot be reduced to a binary distinction between soluble and transmembrane states. Instead, membrane association often reflects regulated transitions among soluble, surface-bound, partially inserted, and deeply embedded conformations (Bigay and Antonny 2012; Burn 1988; Johnson and Cornell 1999).

The membrane itself actively shapes these transitions. Local differences in charge, phosphoinositide content, bilayer thickness, curvature, packing defects, cholesterol content, and phase organization can selectively stabilize distinct protein conformations or binding modes (Drin and Antonny 2010; Phillips et al. 2009; Sezgin et al 2017; Vanni et al. 2014). Regions containing packing defects facilitate shallow insertion of amphipathic helices and hydrophobic loops, whereas ordered or cholesterol-rich environments may preferentially recruit or exclude specific signaling proteins (Drin and Antonny 2010). In this sense, membrane association cannot be interpreted solely as an intrinsic property of protein structure but emerges from reciprocal coupling between protein architecture and membrane biophysics (Bigay and Antonny 2012; Cho and Stahelin 2005; Phillips et al. 2009; Sezgin et al. 2017).

Taken together, these examples indicate that the classical distinction between peripheral and integral proteins should be interpreted as an operational classification rather than as a strict mechanistic boundary. Many membrane-associated proteins occupy intermediate and regulatable positions along a dynamic continuum of interaction states, ranging from transient electrostatic adsorption to shallow monotopic insertion, lipid-anchor-assisted association, mechanosensitive coupling, and stable transmembrane integration. Within this continuum, membrane binding is controlled by the coordinated contribution of protein architecture, post-translational modifications, conformational switches, and membrane physicochemistry. This view provides a flexible basis for understanding how proteins reversibly engage membranes to regulate signaling, trafficking, lipid sensing, membrane remodeling, lipid metabolism, and supramolecular organization.

### Secreted proteins and extracellular modulators

Membranotropism is not restricted to peptides and proteins that reside permanently in membranes. Secreted proteins, extracellular peptides, soluble toxins, amyloidogenic assemblies, annexins, and extracellular vesicles can all modulate membrane structure and function through transient, peripheral, or partially inserted states (Lemmon 2008; White and Wimley 1999; Raposo and Stoorvogel 2013; Butterfield and Lashuel 2010). These systems interact with membranes from the extracellular or luminal side and can alter membrane curvature, lipid packing, domain organization, membrane permeability, and signaling without becoming classical integral membrane proteins (Bigay and Antonny 2012; Marsh 2008b; Phillips et al. 2009).

Secreted modulators use different strategies for membrane engagement. Some rely on transient interfacial anchoring through amphipathic segments, exposed hydrophobic patches, or aromatic-rich motifs to partition into the region between lipid headgroups and acyl chains (White and Wimley 1999). This mode of interaction does not necessarily require stable transmembrane insertion, but it can perturb local packing, generate curvature stress, induce nanoscale defects, and promote transient permeabilization (Bigay and Antonny 2012; Marsh 2008b). Melittin (Bechinger 1997), LL-37 (Henzler-Wildman et al. 2004), and amyloidogenic peptides such as Aβ oligomers illustrate how extracellular or extracellularly exposed peptides can remodel membranes through partial insertion and cooperative surface accumulation (Butterfield and Lashuel 2010). Other systems rely on structured lipid-binding domains to achieve more selective membrane recognition. Annexins, for example, undergo calcium-dependent membrane association with negatively charged phospholipids, especially phosphatidylserine, and can act as scaffolds involved in membrane repair, vesicle trafficking, membrane bridging, and curvature stabilization (Gerke et al. 2005). In damaged membranes, different annexins may accumulate at wound edges, oligomerize (annexin A4), or physically link adjacent membrane folds (annexin A6), thereby promoting rapid membrane resealing and reorganization (Boye and Nylandsted 2016). Several toxins and extracellular signaling proteins also contain lipid-recognition motifs that selectively target cholesterol-, sphingomyelin-, or ganglioside-rich membrane domains prior to membrane insertion, oligomerization, cellular intake, or pore formation. Examples include cholera toxin binding to GM1 ganglioside-enriched membrane domains, which stabilizes membrane attachment and toxin clustering (Merritt et al. 1994), and cholesterol-dependent cytolysins such as perfringolysin O (PFO), which recognize cholesterol-rich membranes before assembling into pore-forming complexes (Rossjohn et al. 1997).

Extracellular vesicles represent a higher-order example of membranotropic architecture. Exosomes, microvesicles, and apoptotic bodies carry proteins, lipids, nucleic acids, and metabolites that can alter membrane composition and cellular signaling pathways (Yáñez-Mó et al. 2015). However, their membranotropic behavior is not restricted to cargo delivery alone. Their own lipid composition, curvature, and surface protein organization influence docking, recognition, hemifusion, and fusion with target membranes (Skotland et al. 2017). Thus, extracellular vesicles can transfer biochemical information while also modifying the physical and compositional state of recipient membranes (Raposo and Stoorvogel 2013; Skotland et al. 2017).

Together, these examples show that extracellular membranotropic systems operate through a broad range of interaction modes, from shallow interfacial anchoring and lipid-specific recognition to scaffolding, vesicle-mediated transfer, membrane remodeling, and pore formation. They expand the architectural spectrum of membranotropism beyond resident membrane proteins, emphasizing that soluble or secreted systems can transiently engage membranes to reorganize their structure, composition, and function.

### Oligomerization and pore formation

Oligomerization is a central mechanism by which membranotropic proteins and peptides convert initial membrane adsorption into complex functional outcomes, including membrane permeabilization, molecular translocation, signaling, and large-scale bilayer reorganization (Cosentino et al. 2016; Mulvihill et al. 2018). Although isolated monomers can associate transiently with membranes through electrostatic attraction or shallow hydrophobic partitioning, many membrane-active processes require cooperative assembly to overcome the energetic barriers associated with bilayer deformation, hydrophobic exposure, and pore nucleation (Huang 2000; Lee et al. 2013). In this context, oligomerization should be viewed not merely as a structural consequence of membrane binding, but as a functional transition that enables membrane-active behavior inaccessible to monomeric states alone.

Following membrane association, proteins and peptides become laterally confined within a quasi-two-dimensional environment at the membrane interface (Wimley 2010). This spatial confinement increases the probability of intermolecular encounters, promotes local accumulation, and favors concentration-dependent cooperative assembly. As a result, membrane activity frequently displays threshold behavior, in which permeabilization or remodeling emerges only after a critical surface density is reached (Huang 2000). Such cooperative transitions have been described for antimicrobial peptides, pore-forming toxins, gasdermins, and amyloidogenic assemblies (Mulvihill et al. 2018; Serra-Batiste et al. 2016; Wimley 2010).

The ability to oligomerize is encoded in structural features that stabilize intermolecular contacts within the membrane environment. Amphipathic α-helices and β-sheet-forming motifs provide complementary hydrophobic and polar interfaces that support peptide–peptide or protein–protein association while maintaining favorable lipid contacts (Cosentino et al. 2016; Hong and Su 2011). Flexible loops, hinge regions, intrinsically disordered segments, and lipid-binding motifs can further facilitate assembly by allowing conformational adaptation, local lipid clustering, or stabilization of membrane defects during pore nucleation and maturation (Epand and Epand 2009; Uversky 2019).

For AMPs and other short membrane-active peptides, three classical models have been widely used to describe membrane permeabilization. In the barrel-stave model, peptides insert approximately perpendicular to the membrane plane and assemble into cylindrical bundles in which the pore lumen is lined mainly by peptide surfaces; alamethicin is a classical example of this mechanism (Qian et al. 2008). In the toroidal pore model, lipid monolayers bend continuously through the pore, so that both lipid headgroups and peptide segments contribute to the conductive pathway, as proposed for peptides such as magainin and melittin (Leontiadou et al. 2007; Matsuzaki et al. 1997). In the carpet model, peptides accumulate parallel to the membrane surface until a critical threshold is reached, leading to detergent-like destabilization and membrane fragmentation rather than discrete pore formation (Wimley 2010). These classical schemes remain useful as conceptual reference points, but many peptide systems can shift among related permeabilization modes depending on concentration, membrane composition, lipid packing, curvature stress, and bilayer tension (Leontiadou et al. 2007; Kabelka and Vácha 2021; Mulvihill et al. 2018; Rakowska et al. 2013).

Proteinaceous pore-forming systems often proceed through more defined oligomeric intermediates. In cholesterol-dependent cytolysins, gasdermins, and MACPF-family proteins, monomers assemble on the membrane surface into prepore complexes before undergoing conformational rearrangements that drive membrane insertion (Gilbert 2002; Mulvihill et al. 2018). Gasdermin-D, for example, can populate arc-shaped, slit-like, and complete ring structures during pore maturation, illustrating how pore formation may proceed through structurally heterogeneous intermediates rather than a single synchronized pathway (Ruan et al. 2018).

Other membranotropic assemblies generate membrane defects without forming canonical enclosed pores. Amyloidogenic β-sheet-rich peptides such as Aβ42 can produce ion-conductive defects through heterogeneous aggregates and partially exposed β-sheet interfaces rather than through well-defined cylindrical channels (Serra-Batiste et al. 2016). Similarly, several AMPs induce transient lipidic defects that assemble and disassemble rapidly, producing stochastic permeabilization events instead of stable pores (Wimley 2010). These examples indicate that pore formation and membrane permeabilization are often ensemble properties of membrane-associated assemblies, shaped by both molecular architecture and bilayer physical state.

The consequences of oligomerization also extend beyond permeabilization. Cooperative assembly can promote localized lipid sorting, membrane thinning, curvature generation, interleaflet coupling, and changes in membrane order, thereby reorganizing the bilayer even in the absence of fully developed pores (Epand and Epand 2009; McMahon and Gallop 2005). Whether oligomerization culminates in transient leakage, stable pore formation, molecular translocation, signaling, or non-lytic membrane remodeling depends on the interplay between intrinsic determinants, such as amphipathicity, conformational flexibility, oligomer geometry, and lipid-binding motifs, and membrane properties, including lipid composition, cholesterol content, charge density, packing, curvature, and tension (Huang 2000).

Taken together, these observations position oligomerization as a central organizing principle in membranotropism. Rather than producing static geometric endpoints, membranotropic proteins and peptides often operate through cooperative assemblies whose structure and function emerge from reciprocal coupling between molecular architecture and membrane biophysics. This perspective shifts pore formation from a purely structural phenomenon to a context-dependent process governed by surface density, transient intermediates, assembly polymorphism, and membrane heterogeneity (Kabelka and Vácha 2021).

## Applications and perspectives

Understanding membranotropism has direct implications for therapeutic design because many membrane-active molecules recognize the physical and chemical state of lipid bilayers rather than a single protein receptor (Escribá et al. 2015). This is especially relevant for antimicrobial strategies, where targeting membrane composition, charge, curvature, or packing may reduce the likelihood of classical resistance mechanisms based solely on receptor mutation (Hancock and Sahl 2006). Rationally designed AMPs such as SAAP-148 and magainin-derived analogues illustrate how tuning cationicity, amphipathicity, hydrophobicity, and stability can improve membrane activity, antibiofilm performance, and selectivity. SAAP-148, a synthetic LL-37-derived peptide, and pexiganan/MSI-78, a magainin-derived analogue, exemplify how structure-guided optimization can enhance membrane disruption and antimicrobial performance, including activity against resistant pathogens and biofilms (de Breij et al. 2018). Together with other peptide-based antimicrobial and antifungal candidates, these examples show that membranotropic design principles have moved from basic membrane biophysics toward translational antimicrobial strategies (Mookherjee et al. 2020).

The same logic applies to antiviral strategies, intracellular delivery, and ion-channel modulation. Viral fusion peptides and transient refolding intermediates of viral fusion proteins have inspired entry inhibitors that block membrane-merging steps, particularly by interfering with pre-hairpin intermediates or six-helix bundle formation (White et al. 2008). Viroporins provide a complementary class of membrane-associated antiviral targets, as their oligomeric ion-channel activity contributes to viral replication, assembly, release, and modulation of intracellular organelle environments (Nieva et al. 2012; Wimley 2010). Membranotropic principles have also been exploited for molecular delivery, as arginine-rich and amphipathic cell-penetrating peptides use electrostatic interactions, transient membrane perturbation, or endocytic pathways to transport cargoes across cellular membranes (Mishra et al. 2011). In ion-channel pharmacology, CRAC channels formed by STIM and Orai proteins illustrate how membrane-associated regulatory interfaces can be targeted beyond direct pore blockade, including the STIM–Orai coupling interface and membrane-proximal gating regions that regulate Ca^2+^ entry (Gudlur et al. 2018, 2020; Prakriya et al. 2006).

Membranotropism also offers opportunities across cancer, thrombosis, inflammation, parasitology, and host–pathogen biology. Lipid-sensing PH domains, K-Ras4B, and MARCKS illustrate how phosphoinositide recognition, lipid nanoclustering, and membrane organization can regulate signaling pathways relevant to cancer and inflammation (Ferguson et al. 1995; Gambhir et al. 2004; Milburn et al. 2003; Sun et al. 2007). Membrane-remodeling and force-coupled systems, including epsin, amphiphysin, integrins, and talin, connect curvature generation, endocytosis, cytoskeletal tension, and mechanotransduction to processes such as trafficking, viral entry, thrombosis, and metastasis (Boucrot et al. 2012; Del Rio et al. 2009; Peter et al. 2004; Zhu et al. 2008). Sterol-dependent platforms and pore-forming systems, represented by caveolin-1, listeriolysin O, and α-hemolysin, further show how cholesterol-rich membrane environments can be disrupted, exploited, or protected depending on the therapeutic context (Köster et al. 2014; Song et al. 1996). In parasitic organisms, MEG-24 and MEG-27 from *Schistosoma mansoni* engage host erythrocyte membranes through distinct mechanisms, including pore formation and surface fluidization, pointing to helminth esophageal proteins as underexplored antiparasitic targets (Felizatti et al. 2020). Similarly, perforin-like proteins from apicomplexan parasites act at critical life-cycle transitions: TgPLP1 contributes to *Toxoplasma* egress (Guerra et al. 2018), whereas PLP1 and PLP2 destabilize erythrocyte membranes during *Plasmodium falciparum* traversal and gametocyte exit (Garg et al. 2013; Wirth et al. 2014). However, translating membranotropic principles into drugs remains challenging. Selectivity must be carefully controlled to avoid host membrane toxicity, and activity can be strongly influenced by serum components, lipid composition, ionic strength, membrane heterogeneity, and local concentration. The translational potential of membranotropism relies on understanding and controlling the interplay between molecular properties, membrane physicochemistry, and biological selectivity. Future strategies will therefore need to integrate sequence engineering, structural modeling, membrane biophysics, and biologically relevant membrane models.

## Emerging concepts: condensate–membrane coupling and mesoscale membranotropism

One emerging frontier in membranotropism is the coupling between biomolecular condensates and lipid membranes. Liquid–liquid phase separation (LLPS) has introduced a new layer of membrane regulation in which proteins or protein-rich assemblies do not simply bind to membranes, but can wet, deform, organize, and mechanically remodel them. Many condensates are driven by multivalent weak interactions involving intrinsically disordered regions, modular interaction domains, nucleic acids, or membrane-associated scaffolds, allowing condensed phases to form at or near membrane surfaces (Hyman et al. 2014). In this context, membranes act as two-dimensional reaction platforms that increase local concentration, orient multivalent interactions, and promote the nucleation or stabilization of condensates at membrane surfaces (Case et al. 2019; Snead and Gladfelter 2019; Snead et al. 2022). In cellular membranes, this two-dimensional concentrating effect occurs within a crowded interface, where protein density and excluded membrane area can further modulate local mobility, encounter frequency, and the nucleation or residence time of membrane-associated condensates (Löwe et al. 2023; Schmitter et al. 2026).

**Fig. 2:**
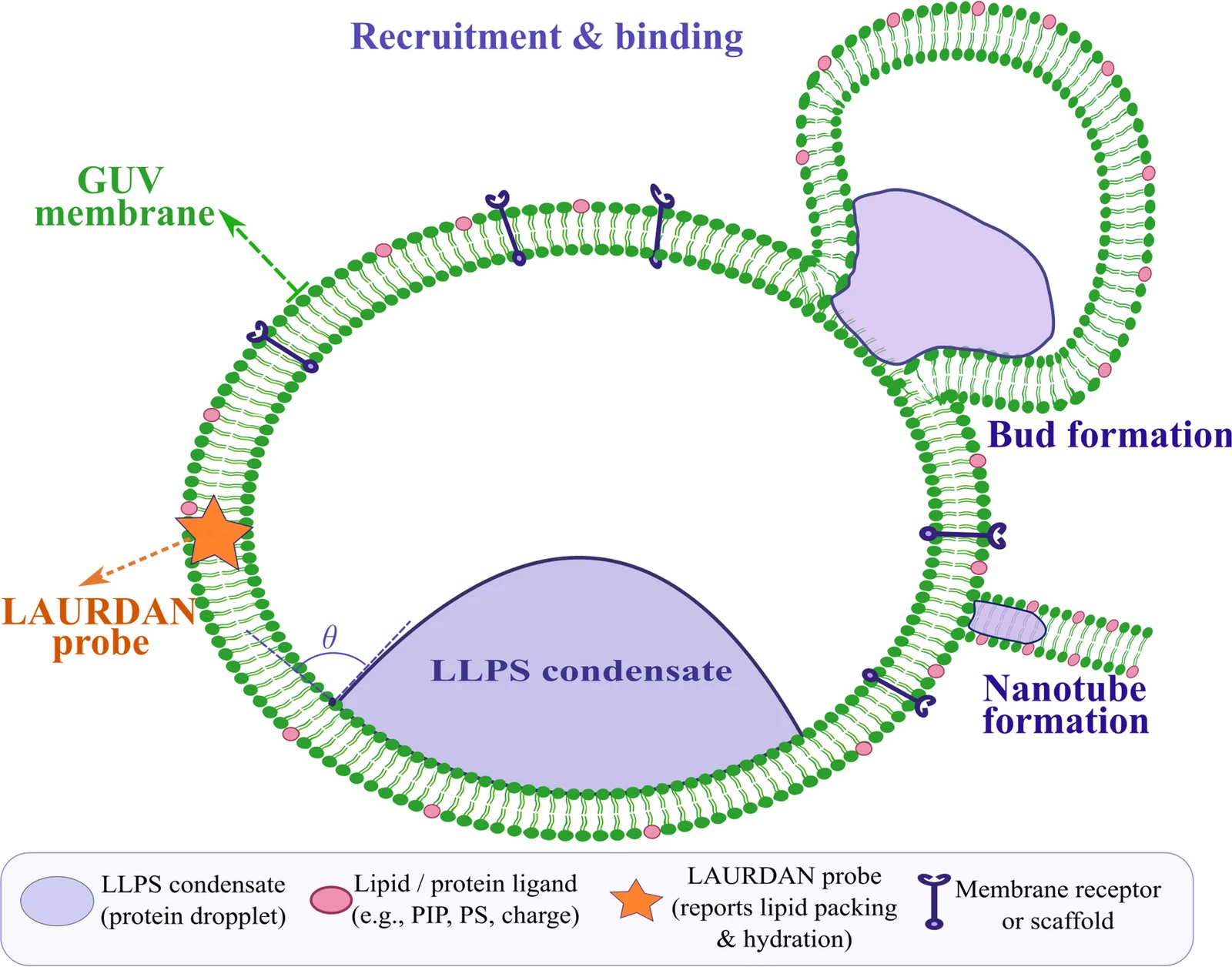
LLPS–membrane coupling in GUV-based experimental systems. Schematic representation of a giant unilamellar vesicle (GUV) interacting with a biomolecular condensate formed by liquid–liquid phase separation (LLPS). Membrane-associated ligands, receptors, or scaffolding proteins can promote multivalent interactions that increase local protein concentration at the membrane surface and favor condensate adhesion. The contact angle *θ* illustrates the wetting geometry of the condensate–membrane interface and can be used to distinguish dewetting, partial wetting, and complete wetting regimes, which depend on parameters such as ionic strength, surface charge, condensate material properties, membrane bending rigidity, adhesion energy, and interfacial tension. Condensate adhesion can locally deform the membrane, promoting budding, ruffling, or nanotube formation along condensate–membrane interfaces. Environment-sensitive probes such as LAURDAN report changes in lipid packing and interfacial hydration associated with condensate wetting. This scheme highlights how biomolecular condensates can act as mesoscale membranotropic assemblies that reorganize membrane structure and mechanics without requiring classical membrane insertion or pore formation. Figure based on Kim et al. 2024; Mangiarotti et al. 2023a, 2023b

Reconstituted systems using giant unilamellar vesicles have shown that condensates can undergo distinct wetting regimes at membrane surfaces, ranging from dewetting to partial and complete wetting, depending on adhesion energy, membrane bending elasticity, interfacial tension, ionic strength, and membrane composition (Mangiarotti et al. 2023a). Membrane wetting by biomolecular condensates has also been associated with the formation of buds, nanotubes, and other curved membrane structures, indicating that condensate adhesion can be directly coupled to membrane deformation (Kusumaatmaja et al. 2021; Mangiarotti et al. 2023a). When excess membrane area is available, condensate adhesion may promote ruffling, budding, tubulation, or curved intermediates reminiscent of membrane remodeling events involved in endocytosis and exocytosis (Fig. 2). These observations indicate that condensates can behave as mesoscale membranotropic assemblies, reshaping membranes without requiring classical insertion, pore formation, or transmembrane integration.

Condensate–membrane interactions can also alter the physical state of the bilayer itself. Fluorescence-based measurements using environment-sensitive probes such as ACDAN and LAURDAN indicate that condensate wetting can modify lipid packing and interfacial hydration, suggesting that the material properties of the condensate phase are coupled to membrane organization (Mangiarotti et al. 2023b). In addition, protein crowding and surface-assisted condensation may generate mechanical or capillary-like effects that influence membrane curvature, domain organization, lipid redistribution, and local membrane tension (Derganc and Čopič, 2016; Yuan et al. 2021). Reconstituted membrane systems have shown, for example, that membrane-associated protein condensates can induce tubules and pearling-like structures in synthetic and cell-derived vesicles, consistent with the idea that interactions within the condensed phase can generate compressive stresses on the lipid bilayer and promote membrane deformation (Yuan et al. 2021). Thus, condensates can modulate membranes not only through biochemical recruitment, but also through physical forces and mesoscale organization.

In cells, membrane-associated condensates are increasingly implicated in signaling, endocytosis, cytoskeletal regulation, and pathological aggregation (Kim et al. 2024). For example, Cu^2+^ was shown to promote condensation of the GPI-anchored prion protein PrP^C^ at the plasma membrane and cell–cell interfaces, linking membrane-associated phase separation to metal-ion buffering, oxidative stress, and pathological protein aggregation (do Amaral et al. 2023). Reconstituted and cellular systems have shown that membrane-associated signaling complexes can undergo LLPS at lipid bilayers, producing condensates that spatially concentrate actin-regulatory or signaling proteins and prolong their membrane residence (Case et al. 2019). Viscoelastic condensates formed by endocytic proteins have also been implicated in membrane remodeling during endocytosis, reinforcing the idea that condensates can participate directly in membrane-shaping processes rather than functioning only as biochemical compartments (Bergeron-Sandoval et al. 2021). This reciprocal coupling between condensate formation and membrane organization expands the concept of membranotropism beyond molecular binding, insertion, and pore formation, linking it to wetting physics, crowding, phase transitions, mechanical force generation, and mesoscale membrane remodeling (Case et al. 2019; Derganc and Čopič, 2016; Snead and Gladfelter 2019).

## Current controversies, knowledge gaps, and future directions

Despite major advances in membrane biophysics, several controversies and knowledge gaps continue to limit a predictive understanding of membranotropism. Classical descriptions of membrane binding, insertion, permeabilization, and pore formation remain useful, but many membrane-active systems populate dynamic and heterogeneous states that do not always fit discrete mechanistic categories. A central unresolved challenge is to determine how observations obtained with simplified membrane models translate to cellular membranes, which are compositionally heterogeneous, asymmetric, crowded, mechanically regulated, and maintained away from thermodynamic equilibrium (Goñi, 2014; Pabst et al. 2026). This issue is especially relevant for processes such as lipid-domain formation, membrane crowding, curvature sensing, peptide-induced permeabilization, and condensate–membrane coupling, where small changes in lipid composition, lateral pressure, leaflet asymmetry, surface charge, membrane tension, or protein density can qualitatively change the observed mechanism. For emerging processes such as condensate–membrane coupling, an additional challenge is to establish how mechanisms identified in reconstituted systems operate within the complexity of living cells (Weakly and Keller 2024; Mangiarotti and Dimova 2024).

Progress will therefore depend on more deliberate use of synthetic and biomimetic membrane systems. Detergent micelles, bicelles, nanodiscs, planar bilayers, vesicles, GUVs, native nanodiscs, and cell-derived membranes each preserve some features of biological membranes while sacrificing others, such as leaflet asymmetry, native lipid diversity, curvature, lateral pressure profiles, crowding, cytoskeletal coupling, or non-equilibrium regulation (Pabst et al. 2026). Rather than treating any single system as intrinsically superior, future studies should select membrane models according to the specific physical property being tested. Controlled synthetic systems with tunable lipid composition, asymmetry, curvature, tension, sterol content, oxidation state, and protein crowding will be particularly valuable when combined with live-cell validation and quantitative biophysical readouts.

Computational approaches are also reshaping the field. Artificial intelligence (AI)-guided peptide and protein design is moving from sequence-based classifiers toward structure-aware and generative strategies capable of proposing backbone and side-chain arrangements with defined physicochemical properties. Diffusion-based methods such as RFdiffusion have already advanced de novo protein design, and their application to short amphipathic, pore-forming, antimicrobial, anticancer, or membrane-binding peptides represents a promising direction (Watson et al. 2023; Brizuela et al. 2025). However, predictive design of membranotropic molecules will require training and validation strategies that explicitly include the lipid environment, because membrane activity is not a property of sequence alone. In parallel, advances in molecular simulations, including multiscale molecular dynamics, enhanced sampling, realistic membrane models, and machine-learned interatomic potentials, are expanding the possibility of following adsorption, folding, insertion, oligomerization, and membrane remodeling with increasing chemical and temporal resolution (Marrink et al. 2019; Eastman et al. 2024). A major future challenge will be to integrate simulation, AI-guided design, and experiment into iterative pipelines that can predict not only membrane affinity, but also selectivity, topology, kinetics, remodeling capacity, and biological outcome.

These conceptual and methodological advances are directly connected to translational applications. Membranotropic agents are attractive candidates for antimicrobial, antifungal, antiviral, antiparasitic, anticancer, and delivery-oriented strategies, but their development is often limited by the gap between in vitro membrane activity and biological efficacy. Antimicrobial and anticancer peptides, for example, frequently show promising activity in simplified membrane assays but face challenges related to serum stability, proteolysis, toxicity, selectivity, delivery, biodistribution, and activity in complex physiological environments (Zheng et al. 2025). Future translational progress will likely require a more integrated workflow in which AI-assisted design, mechanistic simulations, membrane-model selection, quantitative biophysical assays, and cellular or organismal validation are treated as connected steps rather than separate stages. Such integration will be essential for developing predictive models of membranotropism across molecular, membrane, and cellular scales and for designing molecules that recognize, insert into, remodel, or functionally modulate specific membrane environments.

## Concluding remarks

Membranotropism provides a useful conceptual framework that links membrane selectivity, interaction mode, and biological consequence in proteins, peptides, and supramolecular assemblies that engage lipid bilayers. Within this framework, membrane association is viewed as a continuum of interaction states, including surface adsorption, interfacial partitioning, membrane-induced folding, shallow or deep insertion, oligomerization, pore formation, fusion, translocation, curvature generation, lipid clustering, and mesoscale organization. Across biological systems, the same general principle emerges: membrane activity is determined not by a single structural motif or a single lipid property, but by the dynamic matching between molecular architecture and membrane physicochemistry. This matching involves not only lipid composition, charge, curvature, sterol accessibility, packing, and phase behavior, but also lipid oxidation, molecular crowding, membrane mechanics, hydration, and the energetic barriers that govern insertion, fusion, pore formation, and remodeling.

This perspective unifies systems that are often discussed separately, including antimicrobial peptides, viral fusion proteins, ion channels, amphitropic signaling proteins, toxins, parasite-derived membrane modulators, and condensate-associated membrane assemblies. It also highlights an important challenge for the field: predicting membranotropic behavior requires moving beyond static structures and isolated binding events toward quantitative and context-dependent models that incorporate partitioning, residence time, conformational plasticity, membrane heterogeneity, crowding, concentration-dependent assembly, and thermodynamic coupling. In this sense, simplified membrane models remain essential for resolving molecular mechanisms, but their interpretation must be integrated with increasingly realistic synthetic, biomimetic, and cellular membrane systems.

Future progress will depend on combining conceptual, methodological, and translational advances. Complementary experimental approaches, including spectroscopy, calorimetry, scattering, microscopy, structural biology, functional membrane assays, and surface-sensitive methods, together with molecular simulations, are increasingly able to capture transient and heterogeneous membrane-associated states. At the same time, AI-guided peptide and protein design, multiscale simulations, improved membrane models, and cellular validation offer new opportunities to predict and engineer selective membrane recognition and remodeling. By connecting molecular biophysics with membrane physiology, cell biology, infectious disease, and therapeutic design, the study of membranotropism can provide a useful basis for understanding how proteins and peptides recognize, insert into, remodel, and functionally modulate specific membrane environments.

## Data Availability

No datasets were generated or analysed during the current study.

## Declarations

### Declaration on the use of generative AI

During the preparation of this manuscript, the authors used ChatGPT-5.5 as an auxiliary tool to improve language clarity, readability, and overall textual flow. The scientific content, interpretation, and conclusions were conceived, reviewed, and approved by the authors. After using this tool, the authors carefully revised and edited the manuscript and take full responsibility for its final content.
